# Characterization of the cork oak transcriptome dynamics during acorn development

**DOI:** 10.1186/s12870-015-0534-1

**Published:** 2015-06-25

**Authors:** Andreia Miguel, José de Vega-Bartol, Liliana Marum, Inês Chaves, Tatiana Santo, José Leitão, Maria Carolina Varela, Célia M. Miguel

**Affiliations:** Instituto de Biologia Experimental e Tecnológica, Apartado 12, 2781-901, Oeiras, Portugal; Instituto de Tecnologia Química e Biológica, Universidade Nova de Lisboa, Avenida da República, 2780-157, Oeiras, Portugal; The Genome Analysis Centre, Norwich Research Park, Norwich, NR4 7UH, UK; KLÓN, Innovative Technologies from Cloning, Biocant Park, Núcleo 4, Lote 4A, 3060-197, Cantanhede, Portugal; Laboratory of Genomics and Genetic Improvement, BioFIG, FCT, Universidade do Algarve, E.8, Campus de Gambelas, Faro, 8300 Portugal; INIAV- Instituto Nacional de Investigação Agrária e Veterinária, IP, Quinta do, Marquês Oeiras, 2780-159, Portugal

**Keywords:** *Quercus suber*, Fruit, Seed, Transcriptomics, Transcription factor, Transcriptional regulators, Response to water, Carbohydrate metabolism

## Abstract

**Background:**

Cork oak (*Quercus suber* L.) has a natural distribution across western Mediterranean regions and is a keystone forest tree species in these ecosystems. The fruiting phase is especially critical for its regeneration but the molecular mechanisms underlying the biochemical and physiological changes during cork oak acorn development are poorly understood. In this study, the transcriptome of the cork oak acorn, including the seed, was characterized in five stages of development, from early development to acorn maturation, to identify the dominant processes in each stage and reveal transcripts with important functions in gene expression regulation and response to water.

**Results:**

A total of 80,357 expressed sequence tags (ESTs) were *de novo* assembled from RNA-Seq libraries representative of the several acorn developmental stages. Approximately 7.6 % of the total number of transcripts present in *Q. suber* transcriptome was identified as acorn specific. The analysis of expression profiles during development returned 2,285 differentially expressed (DE) transcripts, which were clustered into six groups. The stage of development corresponding to the mature acorn exhibited an expression profile markedly different from other stages. Approximately 22 % of the DE transcripts putatively code for transcription factors (TF) or transcriptional regulators, and were found almost equally distributed among the several expression profile clusters, highlighting their major roles in controlling the whole developmental process. On the other hand, carbohydrate metabolism, the biological pathway most represented during acorn development, was especially prevalent in mid to late stages as evidenced by enrichment analysis. We further show that genes related to response to water, water deprivation and transport were mostly represented during the early (S2) and the last stage (S8) of acorn development, when tolerance to water desiccation is possibly critical for acorn viability.

**Conclusions:**

To our knowledge this work represents the first report of acorn development transcriptomics in oaks. The obtained results provide novel insights into the developmental biology of cork oak acorns, highlighting transcripts putatively involved in the regulation of the gene expression program and in specific processes likely essential for adaptation. It is expected that this knowledge can be transferred to other oak species of great ecological value.

**Electronic supplementary material:**

The online version of this article (doi:10.1186/s12870-015-0534-1) contains supplementary material, which is available to authorized users.

## Background

Seed protection and dispersal are the main functions of the fruit. Fruit initiation and development play a crucial role in plant adaptation, and successful fruiting strategies are important drivers of colonization of new niches. Fruit and seed set are generally characterized by extensive cell division and coordinated development of maternal and filial tissues, while growth and maturation stages are characterized by cell expansion and accumulation of storage products, mainly proteins, starch and oils [1]. The transcriptomic and proteomic analyses of genetic networks operating during specific processes of fruit and seed development have revealed the involvement of a wide range of molecular players including enzymes, regulatory proteins as well as hormonal signals. Molecular studies of fruit development have been mostly conducted in fleshy fruits such as tomato [2–7], grape [8–10], blueberry [11, 12], sweet orange [13] or melon [14], due to their importance for human consumption. Genes related to fruit ripening have been extensively studied in tomato, grape and sweet orange [10, 13, 15, 16] and genes specifically expressed in fruits have been identified in apple [17] and date palm [18]. In addition, *Arabidopsis* has proven very informative because its silique is a dehiscent fruit characteristic of the legumes and thus represents another exceptionally important fruit type in terms of human and animal food.

Much less attention has been paid to other types of fruits that although not generally used for human consumption, have huge ecological importance. The Fagaceae family comprises more than one thousand species half of which belong to the *Quercus* genus, commonly known as oaks. The oaks produce an indehiscent fruit, usually termed acorn, and are characteristically adapted to extremely variable habitats being widely distributed throughout the northern hemisphere in an almost continuous pattern.

Cork oak (*Quercus suber* L*.*), native to the western Mediterranean and north Africa regions [19] characterized by hot and dry summers, has been considered a keystone forest tree species in the ecosystems where it grows [20, 21]. The species is mostly recognized for producing cork, which is removed from adult trees at regular intervals of at least 9 years, sustaining highly profitable cork industries [22]. Cork oak has an unusual fruiting strategy as it is the only known oak species with annual and biennial acorns on the same tree [23–27]. Other features such as bigger acorn size have been related to drought tolerance [28–30] and higher seed germination ability [29–32] and thus may strongly impact the capacity for species natural regeneration, the most common way of cork oak propagation [33]. Seed development and germination are critical for the successful maintenance of the cork oak growing regions [34–36].

During development, the acorn undergoes many biochemical and physiological changes which likely confer the ability to survive the severe drought periods and high temperatures. The few studies that have been conducted in oak acorns have focused on morphological, physiological and phenological aspects [24, 29, 37] and a few reports exist on aspects of the male and female flower development [33, 38–40] and flower/fruit anatomy [41]. Although some transcriptomic and genomic studies have also been published in oak species [42–44], it was only recently that the transcriptome of cork oak has started to be analysed in multiple tissues, developmental stages and physiological conditions [45]. In this context, and to gain knowledge on the molecular mechanisms underlying the development of cork oak acorn and to identify transcripts putatively related with adaptive traits, we have analysed the dynamics of the transcriptome of acorns along five stages of development, defined according to morphological characters, from early fruiting stages to fruit and seed maturation. Our approach identified genes with potentially relevant roles during acorn development focusing specially on transcripts coding for putative transcription factors and transcription regulators or transcripts associated to water related processes including response, transport and deprivation.

## Results

### Categorization of cork oak acorns into different developmental stages

**Fig. 1:**
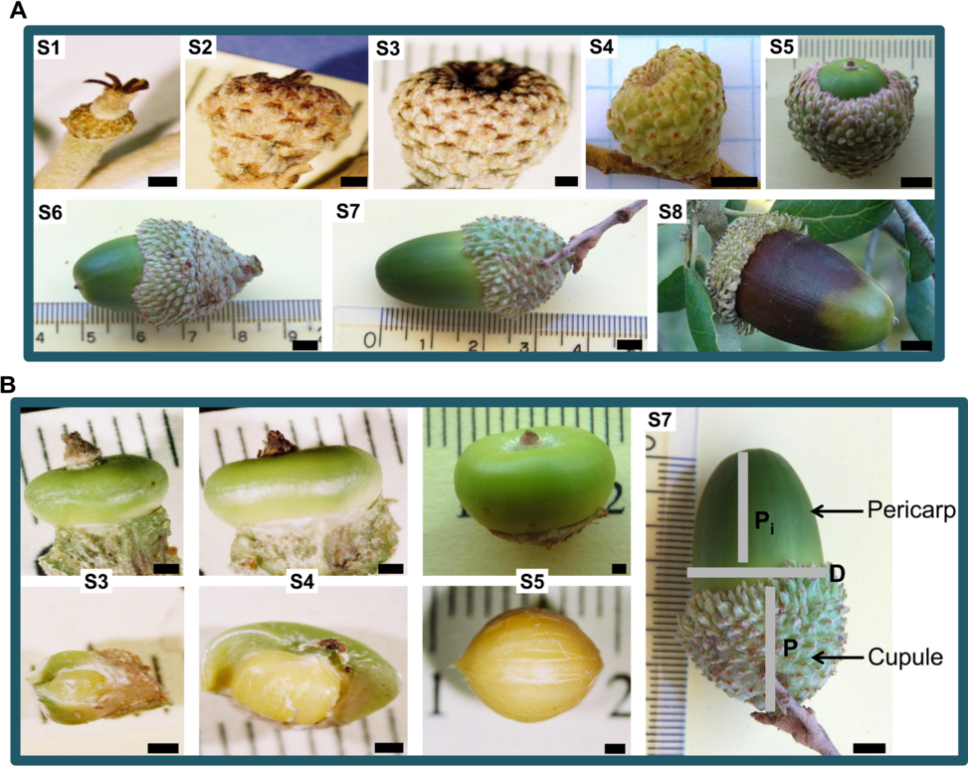
Developmental stages established for the cork oak acorn. **a** Cork oak fruits collected at different developmental stages (S1-S8). The scale bar corresponds to 1 mm in S1 to S3 and to 5 mm in S4 to S8. **b** Cork oak fruits at stages S3-S5 after removal of the cupule (above), or cupule and pericarp (below) exposing the seed, and acorn measurement parameters (S7) used for acorn staging. D, maximum diameter of the acorn; P_i_, portion of the acorn outside the cupule; P, acorn portion covered by the cupule. The scale bar corresponds to 1 mm in S3 to S5 and to 5 mm in S7

Although the fruits are often defined as seed-bearing structures formed from a mature ovary, many structures that might be defined as fruit are in fact composed of different tissue types [46]. Other definitions have been proposed, such as the one by Van der Pijl [47] that considers the fruit as the dispersal unit. In this work we use the term acorn for simplicity, referring in fact to all the tissues enclosed by the pericarp, including the seed. It should be pointed out that at maturity most of the acorn mass consists of seed tissues, mainly cotyledons. Cork oak acorns were collected from late June to November in order to cover all developmental stages, from early development to full maturation. A staging system was established based on several morphological aspects (Fig. 1 and Table 1). Since the dimensions of the acorns were variable in the same collection date among trees in different locations, additional features were used to establish developmental classes. These included the presence of a visible endosperm, multiple embryos or a dominant embryo within the developing seed, covering of the acorn by the cupule and colour of the pericarp (Table 1). Accordingly, eight stages of acorn development were established (S1–S8, Fig. 1a, b). In the first stage (S1), fertilization of the ovules may have occurred already but in most cases the endosperm was not yet visible. In the S2 stage multiple fertilised ovules were visible, however only one continued to grow becoming dominant and causing abortion of the other ovules (S3). During S4 and S5, the embryo continues to develop and in the remaining stages (S6, S7) further enlargement of the cotyledons takes place, with full maturation being reached in S8.

**Table 1:** Criteria used for categorizing the cork oak acorn into different developmental stages. Representation of each stage in the normalized (N) and non-normalized (nN) cDNA libraries. Embryo tissues isolated from acorns belonging to stages S3-S5 and S8 were classified as EM3-EM5 and EM8

Developmental stage	Max Ø with cupule (mm)	Max Ø without cupule (mm)	Isolation of embryos	Other observations; library type
S1	2 - 3	nd	no	no endosperm visible; N and nN
S2	3 - 5	nd	no	multiple fertilized ovules, some aborted; N and nN
S3	5 - 8	nd	yes (EM3)	dominant embryo; N and nN
S4	8 - 12	nd	yes (EM4)	acorn completely covered by the cupule; N and nN
S5	12 - 17	7 - 11	yes (EM5)	acorn already visible out of the cupule; N and nN
S6	17 - 20	11 - 16	no	~1/2 of the acorn out of the cupule
S7	>20	>16	no	Green acorn mostly out of the cupule; N
S8	>20	>16	yes (EM8)	Brown acorn; N and nN

*nd*: not determined

### Sequencing and assembly of the cork oak acorn transcriptome

The sequencing of the five non-normalized libraries corresponding to samples from stages S1, S2, S3 + S4, S5 and S8 aimed at gene expression analysis during acorn development. In addition, two normalized libraries prepared from RNA of cork oak acorns or from isolated embryos were sequenced to favour the detection of rare transcripts and thereby facilitate the assembly. After pre-processing, 2,088,335 high-quality sequences were retained and used in the assembly and mapping steps. The final average length of the reads was 215 and 400 bp for the normalized and non-normalized libraries, respectively (Table 2).

**Table 2:** Read statistics from libraries of cork oak acorn and embryos before and after pre-processing. Embryo tissues isolated from acorns belonging to stages S3-S5 and S8 were termed EM3-EM5 and EM8, respectively

		Non-normalized cDNA libraries					Normalized cDNA libraries	
		S1	S2	S3 + S4	S5	S8	S1 to S8	EM3 to EM5 + EM8
Raw reads	Total	111,703	373,962	200,862	302,253	102,250	738,266	705,151
	Range (bp)	51-1,200	52-1,200	47-1,200	50-1,200	55-1,200	50-1,201	52-1,201
	Mode (bp)	520	521	519	522	519	516	516
	Mean (bp)	503.7	515.8	510.7	524.8	513	538.5	538.7
	Size (Mbp)	47.5	162.5	86.1	133.6	44.3	314.7	298.8
Valid reads	Total	100,081	332,674	179,588	267,171	88,261	572,665	547,895
	Range (bp)	40-568	40-579	40-571	40-565	40-546	40-576	40-616
	Mode (bp)	209	213	220	217	214	409	380
	Mean (bp)	254.1	255.2	253.6	253.5	258.1	304.3	289.2
	Size (Mbp)	27.6	92.7	49.5	74.4	25.5	201.0	183.0

The seven libraries were assembled by MIRA and Newbler (Table 3). MIRA assembly contained 104,862 contigs, 52.2 % of which were longer than 500 bp. Newbler assembly contained 33,034 contigs, 79.6 % of which were longer than 500 bp. The merging of the MIRA and the Newbler assembly using CAP3 resulted in 80,357 contigs that were deposited in ENA (the accession number of the *de novo* transcriptome is [ENA: HABZ01000000] and the accession numbers of the contigs are [ENA: HABZ01000001–HABZ01080357]). 62.5 % of these contigs were longer than 500 bp. The assembled transcripts were classified as complete, terminal, internal or novel by comparison with the complete plant proteins in UniprotKB database (Table 3 and Additional file 1). 23,840 contigs did not have any homologous sequence in the tested database (Complete plant Uniprot proteins). However, it was possible to predict a clear ORF for 4,658 of them, and they were classified as novel.

**Table 3:** *De novo* transcriptome assemblies and classification of the assembled cork oak transcripts

	MIRA	Newbler 2.6	Final (Merged)
Contigs	104,862	33,034	80,357
Contigs > 500 bp	54,764 (52.2 %)	26,313 (79.6 %)	50,197 (62.5 %)
Contigs < 200 bp	6,306 (6 %)	481 (1.5 %)	4,510^a^
Contigs with homologous in Uniprot	73,103 (69.7 %)	26,951 (81.6 %)	56,517 (70.3 %)
Unique Uniprot IDs	28,154 (38.5 %)	16,047 (48.6 %)	24,474 (43.3 %)
Complete contigs	16,149 (15.4 %)	12,953 (39.2 %)	19,146 (23.8 %)
C-terminus contigs	15,818 (15.1 %)	5,342 (16.2 %)	11,410 (14.2 %)
N-terminus contigs	15,368 (14.7 %)	4,064 (12.3 %)	10,108 (12.6 %)
Internal contigs	25,432 (24.2 %)	4,575 (13.8 %)	15,509 (19.3 %)
Misassembled contigs	336 (0.32 %)	17 (0.05 %)	344 (0.43 %)
Contigs without UNIPROT homologous	31,759 (30.3 %)	6,083 (18.40 %)	23,840 (29.6 %)
Novel genes	5,388 (5.14 %)	1,320 (4 %)	4,658 (5.8 %)
Complete ORF	2,758 (2.6 %)	664 (2.01 %)	2,318 (2.9 %)
Partial ORF	2,630 (2.5 %)	656 (1.99 %)	2,340 (2.9 %)
Unknown contigs	26,346 (25.1 %)	4,757 (14.4 %)	19,163 (23.8 %)
Unknown contigs < 200 bp	5,471 (5.2 %)	407 (1.2 %)	0^a^
Putative ncRNAs	25 (0.02 %)	6 (0.02 %)	19 (0.02 %)
Reads mapped^b^	2,020,921 (96.8 %)	1,909,842 (91.5 %)	2,009,759 (96.2 %)
Unique mapped reads	1,703,996 (84.3 %)	1,549,803 (74,2 %)	1,491,131 (71.4 %)
Duplicated mapped reads	273,619 (13.1 %)	270,027 (14.1 %)	367,486 (17.6 %)
Mapping more than two times	43,306 (2.1 %)	90,012 (4.7 %)	151,142 (7.2 %)
Average coverage	7.8	14.2	8

^a^Contigs shorter than 200 bp were filtered out before analyzing

^b^The percentage of reads that mapped over a possible total of 2,088,230 reads

### Completeness of the *Q. suber* transcriptome by comparison with other Fagaceae

The proteins in the *Q. suber* assembly were compared to the proteins from other four *Quercus spp*., two *Castanea spp*. and a *Fagus sp*. For this purpose, we obtained the assembled transcriptomes from the *Fagaceae* project (www.fagaceae.org) or NCBI (for *Q. robur* and *Q. petraea*). The transcripts in each transcriptome were classified as complete, terminal, internal or novel by comparison with the complete plant proteins in UniprotKB database, as we had previously done for *Q. suber* (Additional file 2). Our *Q. suber* assembly had the higher number of complete proteins (19,146) and the second higher number of total proteins (56,517).

On average, 94.2 % of the proteins from any of the tested species could be found in our *de novo* assembled *Q. suber* transcriptome when it was used as the target database (Additional file 3). On the other hand, when the *Q. suber* proteins were used as query, we found that between 60 and 80 % of the queries aligned to each of the other transcriptomes, and the higher ratios corresponded to the more complete *Castanea mollisina* and *Castanea dentata* transcriptomes. Of all the queries, only contig20020 was not found in any other transcriptome.

### Functional annotation of the *Q. suber* transcriptome

All 80,357 transcripts were compared with the NCBI non-redundant (nr) protein database using Blastx with an E-value of 1e^-10^, which resulted in 53,670 (66.8 %) sequences with a significant alignment (Additional file 4). From the total number of transcripts, 19,757 (24.6 %) transcripts had the best match to *Vitis vinifera* sequences, followed by 9,329 (11.6 %); 8,636 (10.7 %) and 5,324 (6.6 %) transcripts that matched to *Ricinus communis*, *Populus trichocarpa* and *Glycine max* sequences, respectively. Similar results have been obtained in the transcriptome annotation for other plant species and linked to conserved biochemical, morphological and developmental characteristics [12]. The number of alignments obtained among the *Fagaceae* family was very low: 122, 101, 97 and 75 sequences matched with sequences of *Castanea sativa*, *Fagus sylvatica*, *Quercus suber* and *Castanea mollissima*, respectively (Additional file 4A). This is mainly due to the limited amount of data available at the GenBank database for non-sequenced species. Most of the alignments showed a similitude between 75 and 90 % (Additional file 4B). Only about 7.6 % of the total number of transcripts present in *Q. suber* transcriptome was found to be fruit or seed specific but, according to our analysis of the conserved motifs and structures in the sequences, the majority of these transcripts are unknown (Additional file 1).

50,228 (62.5 %) transcripts were annotated with at least one Gene Ontology (GO) term (Additional file 5). There was a direct relation between the sequence length and percentage of annotated sequences and over 75 % of the sequences longer than 1 Kb could be annotated (Additional file 4C).

49,945 (52.15 %) *Q. suber* transcripts had a homologous in the *A. thaliana* genome (Blastx E-value < 1e^-10^). Each transcript was annotated with the GO terms of its *Arabidopsis* homologous gene. Additionally, each *A. thaliana* gene was annotated with its NCBI COGs, if any exists, and this annotation was also associated backwards to the original *Q. suber* transcript (Additional file 6). In order to compare our *de novo* transcriptome and identify COGs specific to *Q. suber*, a similar approach was followed for *Q. petraea*, *Q. robur* and the cork oak ESTs database (CODB). 44,300 (55.1 %); 59,572 (74.1 %) and 51,916 (64.6 %) transcripts from *Q. petraea*, *Q. robur* and CODB were homologous to genes from the *A. thaliana* genome, respectively. The distribution of protein clusters is summarized in a Venn diagram (Additional file 7). 2,254 (72.5 %) of a total of 3,110 COGs were present in all the species and 222 COGs were specific to *Q. suber*. Of these 222 COGs, 12.2 % were involved in *replication, recombination and repair*, 6.3 % in *RNA processing and modification,* 5.9 % in *translation, ribosomal structure and biogenesis*, 5.4 % in *cell cycle control*, *cell division and chromosome partition*, 5 % in *post-translational modifications, protein turnover and chaperones* and 5 % in *transcription*. Finally, 20.8 % of the 222 COGs were *unknown* or *poorly characterized* (Additional file 6: File S3 and Additional file 8).

### Pathway analysis during cork oak acorn development

15,612 (19.4 %) sequences were annotated according to their homology with known enzymes that belonged to 140 pathways (KEGG level-3 pathways) and all 14 KEGG groups of related pathways (KEGG level-2 pathways) (Additional file 9). The *carbohydrate metabolism* was the group most represented, which also includes several of the more represented pathways, such as *starch and sucrose*, *glycolysis and gluconeogenesis*, *amino sugar and nucleotide sugar*, *pyruvate*, and *galactose* metabolic pathways. The second most represented group was *amino acid metabolism*, which includes *phenylalanine metabolism*. When the number of different enzymes is considered, the more relevant pathways were *glycine, serine and threonine*; *arginine and proline* and *cysteine and methionine* pathways. The third most represented group was *lipid metabolism* followed by *energy metabolism*. Among the most represented pathways were also *purine* and *pyrimidine metabolism*, *methane metabolism*, and *phenylpropanoid biosynthesis* (Additional file 9)*.*

**Fig. 2:**
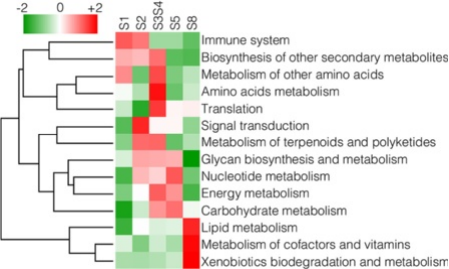
Heatmap of the expression levels of the KEGG level 2 pathways. The expression levels were normalized in Z scores, with signals from red (higher expression) to green (lower expression)

The reads from the five non-normalized libraries were mapped to the transcripts in the final assembly to quantify the expression in each stage. The number of mapped reads of the transcripts belonging to the same pathway was summed up to determine the expression of each pathway on time (Additional file 9). The normalized expression values for the level 2 pathways were represented in a heatmap (Fig. 2). While the *immune system* was the most up-regulated pathway in the first acorn developmental stage (S1), followed by *metabolism of other aminoacids* and *secondary metabolites*, in middle stages of development (S2 to S5) up-regulation of *carbohydrate*, *nucleotide*, *glycan biosynthesis* and *energy metabolism* was observed. *Signal transduction* pathways were up-regulated only in S2, while *amino acid metabolism* and *translation* were specifically up-regulated in S3S4. S8 exhibited an expression profile markedly different from other developmental stages where *lipid metabolism* and *metabolism of cofactors and vitamins* were specifically up-regulated.

### Differentially expressed genes (DEGs) during cork oak acorn development

From the mapping of the reads of the five non-normalized libraries to the assembled transcriptome, 58,839 genes were identified as expressed during any of the developmental stages, 7,824 transcripts (13.3 %) were expressed in all the stages and 22,802 (38.8 %) were specific to one stage. The total number of transcripts present in each stage was 23,104 (39.3 %); 37,501 (63.7 %); 30,035 (51 %); 33,367 (56.7 %) and 17,310 (29.4 %), respectively from S1 to S8 (Additional file 10).

**Fig. 3:**
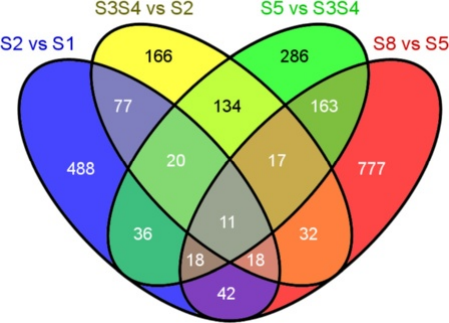
Venn diagram illustrating the number of transcripts differentially expressed between two consecutive stages of development

Of the 58,839 transcripts expressed during acorn development, 2,285 (3.9 %) were considered DE (Additional file 11). From those 710 (31.1 %), 475 (20.8 %), 685 (30 %) and 1,078 (47.2 %) transcripts were DE between stages S2 and S1, S3S4 and S2, S5 and S3S4, and S8 and S5, respectively. 568 transcripts (24.9 %) were DE in more than one stage transition (Fig. 3). From the DEGs only 30 (~1.3 %) were found as acorn specific, with 5 transcripts in stage S1, 14 in stage S3S4, 7 in stage S5 and 4 in the last stage of the acorn development (S8). However, the majority of these transcripts are of unknown function (Additional file 11).

An enrichment analysis by F-fisher test (FDR < 0.05) comparing the set of 2,285 DEGs *versus* the complete transcriptome evidenced 466 over-represented GO terms (Additional file 12). One third of the DEGs were involved in responses to abiotic stimulus, one fifth in carbohydrate catabolism, and other fifth in the catabolism and generation of energetic compounds. GO terms related with transport process, such as water and auxin polar transport, or development and growth were also significantly represented (Additional file 13).

**Fig. 4:**
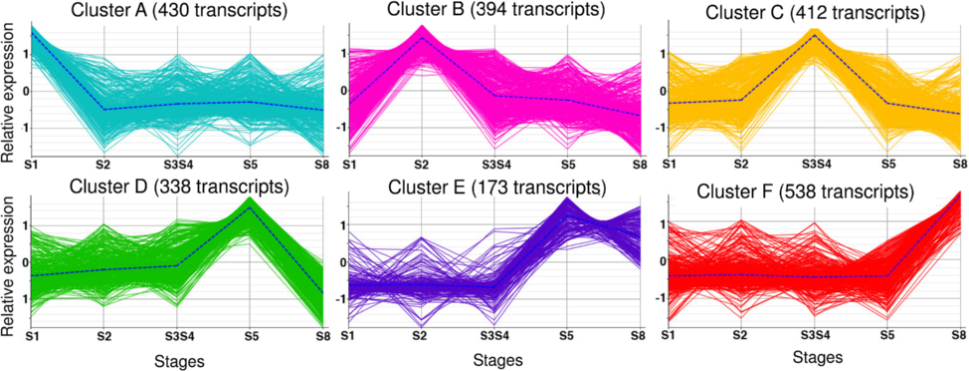
Clustering analysis of differentially expressed genes (DEGs) according to their expression profiles

DEGs were clustered in six groups according to their expression profile on time (Fig. 4 and Additional file 11). Since each cluster contains genes with a peak of expression in specific stage(s) of development, an enrichment analysis (FDR < 0.01) of the genes in each cluster *versus* the complete transcriptome evidenced the dominant processes in those stages (Additional file 12). Eight GO terms were over-represented at S1 (cluster A) including *response to stimulus, response to osmotic* and *salt stress*, and *hexose transmembrane transport*. Forty-one GO terms were over-represented at S2 (cluster B), including *response to stress*, *to water*, *to water deprivation*, *to osmotic* and *to salt stresses*, as well as *water transport*, and *transmembrane trans*port. At S3S4 stages (cluster C) 73 GO terms were over-represented, including the previous terms related with response, and also *regulation of meristem growth* and *of meristem development*, among others. Thirty-one GO terms were over-represented at S5 (cluster D), and 68 GO terms at S5 and S8 (cluster E), including *glycogen synthesis* and *metabolism*, *carbohydrates* (*glucose*, *hexose*, *glucan*) *metabolism*, as well as *starch synthesis* and *xylem development*. Fifty GO terms were over-expressed at S8 (cluster F), including *chitin binding* and *metabolism* and *aminoglycan*, *amino sugar*, *glucosamine* and *polysaccharide catabolism*.

### Genes related to response to water, water deprivation or water transport

From the total DEG, 211 (9.23 %) were related to *water response*, *deprivation* and *transport*, and distributed across all the developmental stages but over-represented in S2, followed by the last developmental stage (S8) (Additional file 14). A shortlist of these transcripts is presented in Table 4, including those that have a known *Arabidopsis* homolog, are specific of a single cluster and have a level of expression at a given stage higher than 85 % compared with its expression in all stages. Fulfilling these criteria and mostly expressed in the first stage of acorn development (S1-cluster A) we found an homolog of the ABC transporter family, *MULTIDRUG RESISTANCE P-GLYCOPROTEINS* (*MDR4*/*PGP4*/*ABCB4*) as well as an homolog of the *UBIQUITIN-CONJUGATING ENZYME 32* (*UBC32*). In cluster B, with a peak of expression in stage S2, an homolog of the *Arabidopsis β-AMYLASE 1* (*BAM1*) was identified. In the subsequent stages of acorn development (S3S4, cluster C) we found a transcript with homology to the *RESPONSIVE TO DESICCATION 22* (*RD22*). Specific to cluster D we found homologs of the *ALPHA-GLUCAN PHOSPHORYLASE 1* (*PHS1*), *EARLY RESPONSIVE TO DEHYDRATION 5* (*ERD5*) and the *ABA INSENSITIVE 3* (*ABI3*). With an expression profile that fits in cluster E, was a member of the *EARLY RESPONSIVE TO DEHYDRATION*, an homolog of the *ERD15*. Transcripts putatively encoding for members of Late Embryogenesis Abundant protein family, such as *LATE EMBRYOGENESIS ABUNDANT 4–5* (*LEA4–5*), *DEHYDRIN XERO 1* (*XERO1*) and *DROUGHT-INDUCED 21* (*DI21*), for *LIPID TRANSFER PROTEIN 3* (*LTP3*), for *URIDINE DIPHOSPHATE GLYCOSYLTRANSFERASE 74E2* (*UGT74E2*) and for *METALLOTHIONEIN 3* (*MT3*) were almost uniquely expressed in the last stage of the acorn development (Table 4 and Additional file 14).

**Table 4:** Shortlist of differentially expressed transcripts annotated as involved in response to water. Transcripts with a known *Arabidopsis* homolog were selected from a total of 211 DEGs in this category, based on their specificity to a single cluster and higher expression level in a given stage as determined by a stage expression factor (SEF^a^) higher than 0.85. To have transcripts specific of cluster D in the short list, the stage expression factor considered in this case was 0.57. Expression in each stage is represented as normalized counts

Cluster	Transcript name	*At* Locus	*At* homologous	Annotation	counts					SEF^a^
					S1	S2	S3S4	S5	S8	
A	Contig16112	AT2G47000	MDR4, PGP4, ABCB4	Multidrug resistance 4, P-glycoprotein 4, ATP binding cassette subfamily B4	19.8	0	0.6	0.4	0	0.95
A	Qs-dev_rep_c84235	AT3G17000	UBC32	Ubiquitin-conjugating enzyme 32	25	0	1.2	0	1.2	0.91
B	Contig18768	AT3G23920	BAM1	β-Amylase 1	1.2	29.6	0.6	1.2	2.2	0.85
	Qs-dev_rep_c76744				0.4	54.2	0.2	8.4	0.8	0.85
C	Qs-dev_rep_c77033	AT5G25610	RD22	Responsive to desiccation 22	0.4	0	34.4	0	3.6	0.90
D	Contig17288	AT3G30775	ERD5, PRODH, ATPOX, ATPDH, PRO1, PDH1	Methylenetetrahydrofolate reductase family protein	0	0	0	22	15	0.59
D	Qs-dev_rep_c76415	AT3G29320	PHS1	Glycosyl transferase	0.4	0.6	0.2	25.4	16.6	0.59
D	Contig19318	AT3G24650	ABI3	AP2/B3-like transcriptional factor family protein	0	0	7.2	66.8	42.2	0.57
E	Contig19981	AT2G41430	ERD15, LSR1, CID1	Dehydration-induced protein (ERD15)	0	2.2	0.6	25.8	37.2	0.96
F	Contig18471	AT5G59320	LTP3	Lipid transfer protein 3	0	0.2	0	5.6	38.6	0.87
F	Contig16935	AT4G15910	DI21	Late embryogenesis abundant protein Lea5; drought-induced 21	1.2	0	3.6	18.8	179.2	0.88
	Qs-dev_c42419				0	0	0	0	16.4	1
F	Contig18808	AT5G06760	LEA4-5	Late embryogenesis abundant 4-5	1.2	2.2	0.6	0.4	53	0.92
F	Contig17917	AT3G50980	XERO1	Dehydrin xero1	3	0.8	0.6	3.4	50.8	0.87
F	Contig19993	AT1G05680	UGT74E2	Uridine diphosphate glycosyltransferase 74E2	0	0.8	0	4.2	33.8	0.87
F	Qs-dev_rep_c73281	AT3G15353	MT3	Metallothionein 3	0.4	3	0.2	4.6	75	0.90
	Qs-dev_rep_c79475				0.4	0	0.2	0.8	18.8	0.93

^a^ $$ Stage\  Expression\  Factor=\frac{normalizedcount{s}_{stageX}}{{\displaystyle \sum } normalizedcount{s}_{allstages}} $$

### Transcriptional regulators differentially expressed during acorn development

Transcription factors have important roles in gene expression due to their ability to bind specific DNA sequences and control transcription by acting as transcriptional activators or repressors. Out of 2,285 DEGs during acorn development a total of 498 (21.8 %) were annotated as TFs or transcriptional regulators (Additional file 15). These transcripts were almost equally distributed among the different clusters, but slightly up-regulated in early development, representing approximately 22.5 and 20.7 % of the transcripts in clusters A and B, respectively, and less expressed in the late stages of acorn development representing 7.4 and 15.9 % of the total DE TFs in clusters E and F, respectively.

A list of selected transcripts that have a known *Arabidopsis* homolog and are annotated as transcription factors or transcriptional regulators is presented in Table 5, including those that are specific of a single cluster and are also stage-specific or belong to TF families with well characterized roles in plant development. We found homologs of *MYB DOMAIN PROTEIN 36* (*MYB36*) and *AUXIN RESPONSE FACTOR 4* (*ARF4*) specifically expressed in stage S1 (cluster A) and a member of the MYB-related family, homolog of the *PEROXIDASE 72* (*PRX72*) specifically present in stage S3S4 (cluster C). Also other transcripts were identified which expression was restricted to the late stages of development, such as a homolog of the *FAR1-RELATED SEQUENCE 4* (*FRS4*) in stage S8 (cluster F). Interesting genes from well-known families of TFs or transcriptional regulators such as NAC, bHLH, class II *KNOTTED1*-like homeobox and OLEOSIN are also represented during the cork oak acorn development. Up-regulation in the early stages of development (S1 and S2) was observed for class II *KNOTTED1*-like homeobox genes. During the late stages of the acorn development transcripts putatively coding for *OLEOSIN* (*OLEO*) were up-regulated. Transcripts coding for NAC and bHLH transcription factor families were found DE across all the studied developmental stages (Table 5 and Additional file 15).

**Table 5:** Shortlist of differentially expressed transcripts putatively coding for transcription factors and transcriptional regulators. Transcripts with a known Arabidopsis homolog were selected according the following criteria: transcript is either specific of a single cluster and is stage-specific or belongs to TF families with well characterized roles in plant development

Cluster	Transcript name	Transcription factor	Domain /Family	*At* Locus	*At* homologous	Annotation	counts					SEF^a^
							S1	S2	S3S4	S5	S8	
A	Contig20395	Vitis_vinifera_ GSVIVP00000629001	MYB_DNA binding/MYB	AT5G57620	MYB36	MYB domain protein 36	24.2	0	0	0	0	1
A	Contig14583	Vitis_vinifera_ GSVIVP00024505001	B3/ARF	AT5G60450	ARF4	Auxin response factor 4	21.6	0	0	0	0	1
C	Contig20996	Oryza_sativa_subsp._Indica_OsIBCD010288	MYB_DNA binding/MYB-related	AT5G66390	PRX72	Peroxidase superfamily protein	0	0	16.2	0	0	1
F	Contig14504	Oryza_sativa_subsp._japonica_LOC_Os11g45530.1	FAR1/FAR1	AT1G76320	FRS4, CPD25	FAR1-related sequence 4	0	0	0	0	23.2	1
A	Contig23988	Carica_papaya_emv.TU.supercontig_187.28	KNOX1/HB	AT5G25220	KNAT3	KNOTTED1-like homeobox gene 3	35.6	0.8	3.8	11.6	13.8	0.54
B	Contig17627	Vitis_vinifera_ GSVIVP00005632001		AT5G11060	KNAT4	KNOTTED1-like homeobox gene 4	1.2	34	27.4	5.6	2	0.48
E	Contig24728	Carica_papaya_emv.TU.supercontig_996.1	AP2/AP2-AREBP DUF260/LOB	AT4G25140	OLEO1	Oleosin 1	0	0	3	69.2	58.5	0.98
E	Contig20473	Vitis_vinifera_ GSVIVP00016010001					0	0	8.2	428.2	192.4	0.99
E	Contig23293	Vitis_vinifera_ GSVIVP00016010001	DUF260/LOB	AT3G27660	OLEO4	Oleosin 4	0	0	0	71	25.8	1
F	Qs-dev_rep_c103605	Vitis_vinifera_ GSVIVP00016010001					0.4	0	0	9.2	39.6	0.80
F	Qs-dev_c70574	Vitis_vinifera_ GSVIVP00016010001		AT5G40420	OLEO2	Oleosin 2	0.2	0	0	2.2	49.8	0.95
B	Contig1681	Carica_papaya_evm.TU.supercontig_3.229	HLH/bHLH	AT5G08130	BIM1		0	22.8	0	1.2	8.8	0.70
D	Contig25637	Vitis_vinifera_GSVIVP00008627001		AT1G01260	JAM2	Jasmonate associated MYC2 like 2	5.2	16.6	7.4	21.6	0	0.43
E	Contig18945	Populus_trichocarpa_180347		AT5G41315	GL3, MYC6.2	Glabra 3, Glabrous 3, MYC6.2	0	4.6	0.6	24.6	23.8	0.90
A	Contig4505	Populus_trichocarpa_218417	NAM/NAC	AT1G56010	NAC1	NAC domain containing protein 1	34.2	2.2	0.6	0	0	0.92
A	Contig23086	Populus_trichocarpa_714847		AT1G34190	NAC017, ANAC017	NAC domain containing protein 17	20.2	0	0	11	4.6	0.56
B	Contig23085						0	27.8	22.8	18.4	7	0.37
A	Contig16452	Vitis_vinifera_ GSVIVP00027622001		AT4G27410	RD26, ANAC072	Responsive to desiccation 26	608.8	184.2	138.6	32	11	0.62
B	Contig25368	Vitis_vinifera_ GSVIVP00000471001		AT4G35580	NTL9	NAC transcription factor-like 9	18.2	42	8.2	14.8	0	0.50
D	Contig26025	Vitis_vinifera_ GSVIVP00027621001		AT3G15510	NAC2	NAC domain containing protein 2	1.2	1.6	12	19.8	0	0.57

^a^ $$ Stage\  Expression\  Factor=\frac{normalizedcount{s}_{stageX}}{{\displaystyle \sum } normalizedcount{s}_{allstages}} $$

### Validation of the differential expression profiles by RT-qPCR

**Fig. 5:**
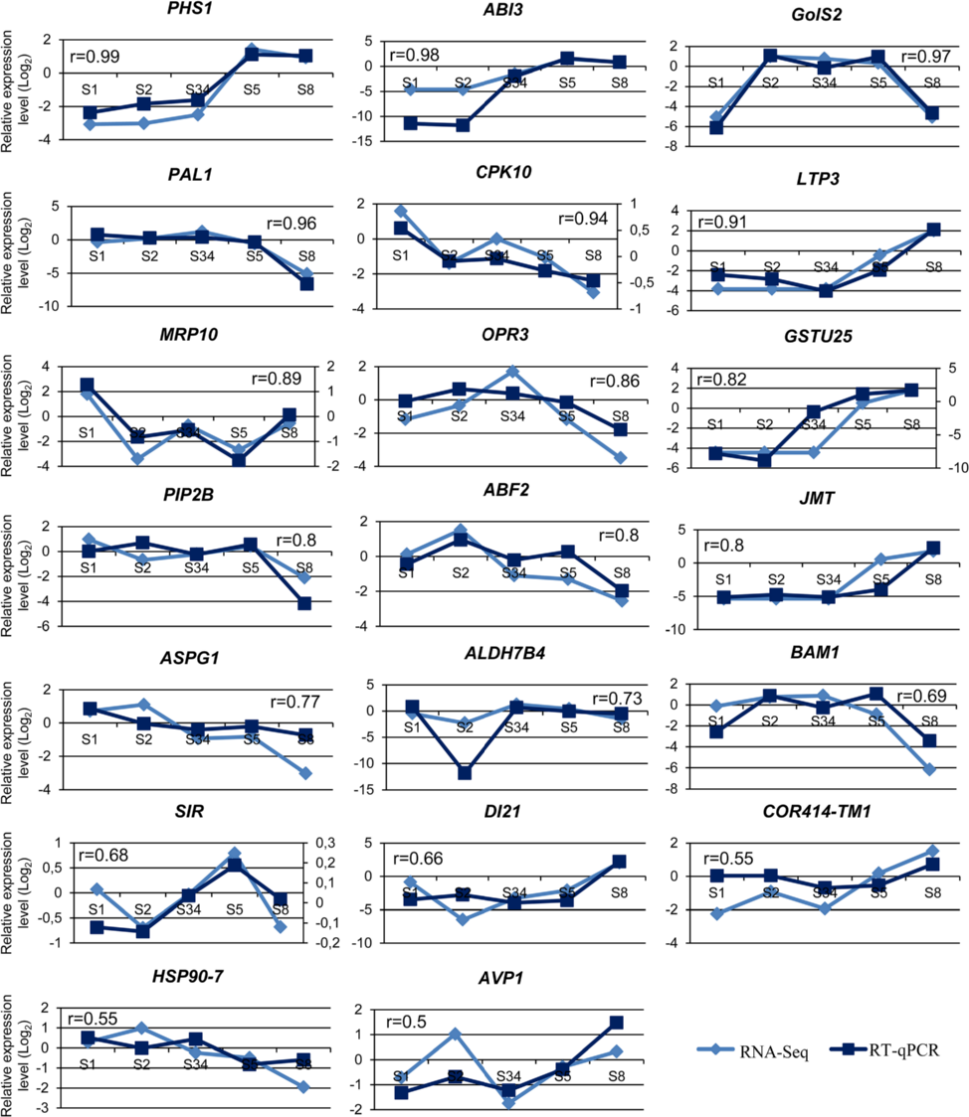
Validation of the RNA-Seq transcript profiles. Comparison of transcripts expression patterns from RNA-Seq data and from reverse transcription quantitative real-time PCR (RT-qPCR). In the y-axis it is represented the Log_2_ of the relative expression level in each developmental stage and the five acorn developmental stages are represented in the x-axis. The numbers above the graphics correspond to the values obtained with the Pearson correlation

Several genes were selected to validate the data obtained by sequencing with 454 Technology (Table 6). Twenty DEGs related to water responses, seven of which also annotated as TFs, were chosen for the validation of expression profiles by reverse transcription quantitative real-time PCR (RT-qPCR). Two transcripts belong to cluster A, six to cluster B, three to C, four transcripts belong to D and five to F. Correlation between the gene expression levels and the profiles obtained by 454 technology was demonstrated by Pearson correlation (Fig. 5) with most of the genes showing strong or moderately strong correlation [48]. In addition, these results also validate that the transcript assemblies are correct for the sequences tested and support the robustness of the transcriptome assembly performed in this work.

**Table 6:** Primers used in RT-qPCR for validation of the expression profile obtained by 454 sequencing.

Cluster^a^	Gene abbreviation^b^	Gene description	Transcript name	*At* Locus	Primer sequences (forward / reverse)
A	MRP10, ABCC14	ABC transporter C family member 14	Contig3296	AT3G62700	GCTGCCTTTGCCCCACACT/ TGGAAGAGCCTTGAACGCTGCC
A	CPK10, CDPK1	Calcium-dependent protein kinase	Contig4133	AT1G18890	GCTCACCTCCCTCGCACAACT/ AGGCGCGAGGTGGCGATAAT
B	ASPG1	Protein aspartic protease in guard cell	Contig19387	AT3G18490	ATGCAGCTCGCTCGACGTGT/ TGTCGTGTCCGCAACCCAGA
B	ABF2	Abscisic acid responsive elements-binding factor 2	Contig22999	AT1G45249	CGGCCTGCTTTTTCCGCAACT/ AGTCAGCTGCAAGGTCACGAGC
B	AVP1	Pyrophosphate-energized vacuolar membrane proton pump	Contig 8793	AT1G15690	CGCACTTGAGAACGACGCT/ TGCGCGGTCGTCGGAATCAT
B	HSP90-7	Chaperone protein htpG family protein	Qs-dev_rep_c72408	AT4G24190	CAGCATCAGCTTCAGCCTCGGT/ AGCGGCTTCACGCCTTCTGA
B	BAM1, BMY7, TR-BAMY	β-amylase 1	Contig 25491	AT3G23920	AACGCGAGTTTGCAGGCGCT/ CGACGTTTCCGCCGCATTGA
B	GolS2	Galactinol synthase 2	Contig 17943	AT1G56600	TGACGCCATAACATGGCCAGCA/ TTGCCAGCAGTGCCCTGACA
C	ALDH7B4	Aldehyde dehydrogenase 7B4	Contig 9124	AT1G54100	AGGGTGCTCCAACGACTCCA/ ACGCACAGCTAGGCCGATG
C	OPR3	Oxophytodienoate-reductase 3	Contig 16616	AT2G06050	TTGGTCTCTAACACAGCCGCCG/ CACGGCCGACATGCCATA
C	PAL1	Phenylalanine ammonia-lyase 1	Contig 9321	AT2G37040	TGCTAACTGGCCGCCCCAAT/ GCCAGAACCAACAGCTGTGCCA
D	ABI3, SIS10	Abscisic acid insensitive 3	Contig 19318	AT3G24650	GCAGTGGCCGTGGTGCAAT/ ATGGCGAGGCAAAGGCGGTT
D	PHS1	Glycosyl transferase, family 35	Contig 10143	AT3G29320	TGGCTTGAGATGGGCAACCCT/ TTTGGTGTGCTCCCCGGCAT
D	SIR	Sulfite reductase	Contig 19883	AT5G04590	TGCAATGGCATGCCCAGCCT/ ACTGGGGTTTCCACCAAGCCA
D	PIP2B	Aquaporin PIP2-4	Contig 24062	AT2G37170	GCTTGGGCCTTTGGTGGCA/ TGGCTCCACCACCGTACTTGCT
F	COR414-TM1	Cold-regulated 314 inner membrane 1	Contig 19349	AT1G29395	TGGTGAGCTTGAGCTGCCGT/ TGCAAGCTGCCTGCTCGTGA
F	DI21	Late embryogenesis abundant protein	Contig 16865	AT4G15910	AGCGTGCTCTCACCTTGTGGT/ TGCGGCTGCATCACATGCCT
F	JMT	Jasmonic acid carboxyl methyltransf.	Contig 17776	AT1G19640	TGGCTATCTTCGCCGCCCTT/ TGCCGTGGTTTGCACCTACTTCG
F	GSTU25	Glutathione S-transferase TAU 25	Contig 17981	AT1G17180	ACCACCTCGTCCGCCATTGT/ AGCCTGACCGACTCCATGGCAA
F	LTP3	Lipid transfer protein 3	Contig 19993	AT5G59320	TGCCCAGGCCACCATAACATGC/ TGTGGTCTTGGCCATGCCGTT

^a^ According to their expression profiles each transcript belongs to a different cluster

^b^ Corresponds to the homologous in *Arabidopsis thaliana*

## Discussion

Based on available genomic resources and NGS technologies we provide here the first overview of the dynamics of the transcriptome along different stages of the acorn development in a Fagaceae species. The analysis of our data highlighted specific genes and processes relevant to better understand the molecular mechanisms involved in cork oak acorn development, and it is expected that this knowledge can be transferable to other oak species of great ecological value. The studied stages of development were established according to morphological criteria and to previously described reports on cork oak reproductive features [33] in order to cover the whole fruit and seed developmental process.

A set of 2,285 differentially expressed genes were identified with roles in a range of biological processes. We then focused our analysis on groups of transcripts with putative functions in transcriptional regulation and traits likely relevant to seed survival and dispersal, including mechanisms related to water response, water transport and water deprivation.

### A *de novo* transcriptome of cork oak acorn

A *de novo* transcriptome assembly with the data here generated allowed us to identify the transcripts expressed during the acorn developmental process, some of which classified as novel. This *de novo* assembly facilitated the mapping of reads in unique positions since it was not necessary to allow mismatches between reads and reference. In fact, we discarded the marginal number of reads mapping in several positions. Assemblers of 454 transcriptome data have been systematically compared using real and simulated datasets. In such reviews, Newbler [49] and MIRA [50] outperformed other assemblers [51–54]. Newbler usually assembles longer contigs that often cover more than the 80 % of the reference sequences. MIRA joins reads in a more conservative way than Newbler, which prevents chimeric contigs and generates bigger assemblies using more bases and containing higher number of contigs, but some of them are redundant. Kumar *et al*. [52] proposed an assembly strategy that was used for the *de novo* assembly of pyrosequencing data from chickpea [53], by merging individual assemblies using a traditional Overlap-Layout-Consensus assembler, such as CAP3 [55]. Merged datasets aligned better to reference datasets and were more consistent in the total span and number and size of contigs than individual assemblies. In our case, the number of complete contigs (19,146) was higher in the merged assembly than in the individual ones, while the percentage of C-terminal and N-terminal contigs was smaller in the merged assembly than in any of the original assemblies (Table 3). This supports that several contigs from the same transcript were merged. When compared with other Fagaceae transcriptomes (Additional file 2), we report the highest number of complete proteins and different unique Uniprot IDs, which evidenced the advantages of this strategy.

Pathway analysis revealed that *carbohydrate metabolism* was the group (KEGG level-2 pathway) most represented in the transcriptome of developing cork oak acorns, especially in the middle stages of development. The enrichment analysis performed in the different clusters evidenced also the timing when a specific metabolic process appears prevalent. Using this approach, carbohydrates metabolism and starch synthesis, were found over-represented in the transcriptome of acorns at late stages of development, both S5 and S8. However, specific processes like hexose transmembrane transport were found over-represented in early stages of acorn development, where actively dividing cells contribute to a rapid growth of the fruit. In general, hexoses favour cell division and expansion, whereas sucrose favours differentiation and maturation [56]. This is also supported by the analysis of DEGs. For instance during the middle stages of acorn development, several up-regulated DEGs homologous to *SUCROSE SYNTHASES* were identified which are putatively involved in the synthesis of UDP-glucose and ADP-glucose linked to cellulose and starch biosynthesis [57]. These include *SUCROSE SYNTHASE 3* (*SUS3*), and *SUCROSE SYNTHASE 4* (*SUS4*). This is consistent with an active synthesis of cellulose and starch during these developmental stages, possibly related to the mobilization of sucrose into pathways involved in structural and storage functions. One fifth of the identified DEGs are related to carbohydrate metabolism and some of these transcripts, involved in water response or transcriptional regulation, are discussed below.

### Response to water across acorn development

At complete maturity cork oak acorns contain a large and fleshy embryo with high water content. The natural shedding of cork oak acorns coincides with complete maturity and acorns left on the ground after shedding will either germinate or lose their viability as a result of desiccation [24]. Increased tolerance to desiccation may thus represent an important factor in cork oak regeneration success and the identification of transcripts related to response to water, water deprivation or water transport may prove relevant for highlighting genes with adaptive roles. In agreement with previous reports in *Arabidopsis* [58, 59], the DEGs annotated as being related to water responses during acorn development are not fruit specific. A high number of DEGs in this category were identified in the cluster of transcripts with a higher expression at the last stage of acorn development corresponding to maturity, probably reflecting some reduction in water content at this stage, but also during the early stages. The early stage of fruit and seed development is one of the most sensitive periods of the plant life cycle to abiotic stresses such as drought [60]. Therefore, it is not surprising that transcripts involved in response to water stress are up regulated at these stages as to ensure the protection of offspring against conditions of low water availability common during the summer.

Among the transcripts strongly related to drought stress that were identified in early development are several homologs of the RD family, including *RD22* and other members not listed in Table 4 such as *RD19*, *RD21, RD26* and *RD29B. RD22* and *RD26*, were previously described to respond to dehydration [61–64] and used as drought-induced marker genes in different plant species [65–69]. *RD22* is expressed in the early and middle stages of *Arabidopsis* seed development [62] and in our transcriptome analysis it is also highly expressed in the middle stages of the acorn development (S3S4, cluster C). During the early stage of the acorn development (cluster A), a putative homolog of *MDR4* annotated as being involved in response to water deprivation was also highly expressed. MDR4 belongs to the large family of the ABC (ATP binding cassete) transporter superfamily and has been mainly characterized for its role in the basipetal redirection of auxin from the root tip [70, 71]. With the same expression profile (cluster A) was a putative homolog of *UBC32*, highly induced under drought stress [72]. Recently, functional studies in tomato revealed that UBC32 is involved in the regulation of fruit ripening [73] and, it is possible that it plays a similar function in cork oak acorn development. Carbohydrate metabolism-related transcripts with homologies to *β-AMYLASE* genes, which are described as contributing to osmoprotection during desiccation [74], were also found differentially expressed during acorn development. While some of the amylase transcripts, e.g. homologous to the *A. thaliana* chloroplast *β-AMYLASEs BAM1/BAMY7*, were up-regulated from the early to middle stages, other transcripts with homology to *β-AMYLASEs BAM5* or *BAM6* were up-regulated in later stages up to S8, being almost absent in earlier stages. The significance of these divergent expression patterns is not currently clear. It is possible that different roles related for instance to hormone signalling and/or acquisition of desiccation tolerance involving the accumulation of soluble sugars through starch degradation [75] are played by these enzymes during seed development.

Specific to cluster D are two transcripts putatively related to abscisic acid (ABA) signalling/responses, homologs of *PHS1* and *ABI3. PHS1* is a negative regulator of ABA signalling involved in the regulation of stomatal aperture [76]. Its expression pattern under different stresses vary significantly among species but it has been associated to drought responses [77]. Like in barley, we detected low expression of this transcript in early acorn development and its putative involvement in the regulation of starch synthesis during endosperm development [77, 78] may explain its up-regulation in later stages of seed development (S5). With the same expression profile is a homolog of a well characterized gene related to acquisition of tolerance to seed desiccation, *ABI3*, putatively encoding a TF expressed in seeds that mediates ABA responses. In *Arabidopsis*, *ABI3* is required during seed maturation for the accumulation of seed storage proteins, dormancy onset and maintenance and for the acquisition of seed desiccation tolerance [79, 80]. Our results seem in agreement with a tight regulation of the mechanisms controlled by *ABI3* during cork oak seed development towards maturation, such as ABA responsiveness and synthesis and accumulation of proline in order to increase stress tolerance in the embryo [81]. The expression profile of *ABI3* seems consistent with alterations in ABA content as described for the recalcitrant seeds (desiccation-sensitive) of *Quercus robur* [82, 83] and corresponding ABA-induced processes along development. Like *PHS1* and *ABI3*, an homolog of the *EARLY RESPONSIVE TO DEHYDRATION 15 (ERD15*) is up-regulated in stage S5 (cluster E). *ERD* genes are rapidly activated by dehydration and the encoded proteins constitute the first line of defense against drought stress [84, 85]. In *Arabidopsis ERD15* is a common regulator of the ABA response and salicylic acid (SA)-dependent pathway [86] encoding a protein with functions in stress responses, including drought [87].

During the last stage of acorn development (S8, cluster F) several transcripts protrude from the list of DEGs annotated as related to water responses. A homolog of *LTP3*, known in *Arabidopsis* to be highly expressed in mature siliques and induced by drought [88, 89], was specific to the late stages of acorn development. The *Arabidopsis LTP3* positively regulates the plant response to drought stress through the transcriptional activation of the cuticular wax biosynthesis to avoid dehydration [88, 89]. Also well represented in cluster F are the *LATE EMBRYOGENESIS ABUNDANT* (*LEA*), the protein family to which *DI21* belongs, as well as *XERO1* and *LEA4–5*. This protein family plays major roles in desiccation tolerance [90] and is characteristically accumulated in the last stages of seed development [91] as also observed in our data. The *Arabidopsis XERO1* was only detected in seeds whereas *DI21* was detected ubiquitously [92]. *LEA4-5* highly accumulates during late *Arabidopsis* embryogenesis and in dry seeds, and upon constitutive expression it confers tolerance to severe drought [91]. In oil palm *LEA4* is solely expressed in the mesocarp, and similarly to what we have obtained in the cork oak acorns, it is expressed in the late stages of fruit development, being probably involved in plant adaptation and stress (drought) responsive pathway [93]. Also *DI21* is known to be up-regulated under abiotic stresses and *XERO1* is thought to protect cellular components from dehydration stress [92]. Accumulation of LEA proteins during seed maturation and in response to altered water status was previously observed in oak species [82, 94, 95]. However, their effect in increasing drought tolerance in oaks is not as extensive as in non-recalcitrant seeds [83]. Another worth mentioning transcript putatively related to desiccation tolerance and up-regulated in the last stage of the acorn development is a homolog of the *UGT74E2*, encoding a hydrogen peroxide–responsive UDP-glucosyltransferase. Ectopic expression of *UGT74E2* was associated to changes in plant architecture and to an increased tolerance to drought and salt stresses [96]. Increased tolerance to abiotic stresses, such as drought, was also verified in transgenic tobacco plants overexpressing the cotton type 3 metallothionein (*GhMT3a*) [97], a homolog of which was also identified up-regulated in late acorn development. Interestingly, many members of this group are associated with fruit ripening [98–101]. Like in the cork oak acorns, also in apple and kiwifruit *MT3* was barely detected in young fruits but accumulated later with fruit development and fruit ripening [98, 100] suggesting a role in the ripening process, as well as in setting drought tolerance in this phase of development.

### Transcriptional regulators during acorn development

Transcriptional regulators are crucial for plant developmental processes through their function in the regulation of gene expression, and fruit and seed development is no exception. In *Arabidopsis* which, like cork oak, has dry fruits, it has been revealed that the core and extended genetic network controlling fruit development consists entirely of interactions among transcription factors [46]. In fact, we found a significant number of differentially expressed transcripts coding for putative transcription factors and other transcriptional regulators along the acorn developmental stages covered by our analysis. However, our results support a slightly more prominent role of these transcripts in early development (S1 and S2) becoming less represented towards acorn maturity. In fact, it is during this stage that the transcription machinery seems more active as evidenced by the higher number of expressed transcripts.

Genes of the auxin response factor family (*ARFs*) are key regulators of auxin-modulated gene expression and can either activate or repress transcription of auxin-responsive genes [102]. A putative ortholog of *ARF4* was found specifically expressed in cluster A, suggesting an important role in the initial phase of the acorn development. In tomato, *ARF4* was described to repress the expression of auxin-responsive genes, playing a key role in the control of sugar metabolism during fruit development, through the regulation of photosynthetic activity as well as chlorophyll and starch accumulation [103]. Moreover, it was found highly expressed in the pericarp tissues of immature fruit and then undergoing a marked decline at the onset of ripening associated with the increase in sugar content accumulation [103]. It is possible that the transcript identified in our data has similar functions in the cork oak acorn development. Interestingly, *ARF4* is a putative target gene of ta-siRNAs produced from cleavage of the ta-siRNA locus 3 (TAS3) directed by miR390 which was reported as differentially expressed in cork oak tissues [104].

Two putative class II *KNOTTED1*-like homeobox (*KNOX2*) genes were also up-regulated in the early stages of acorn development, one in S1 (*KNAT3*) and the other one in S2 (*KNAT4*). *KNAT3* was previously reported to have a role in seed development, specifically in embryo sac development and during megagametogenesis [105]. More recently, KNOX2 TFs were shown to have a critical role in establishing an alternation of generations in land plants by preventing the haploid-specific body plan from developing in the diploid plant body [106], which appears consistent with the up-regulated expression of cork oak putative *KNOX2* genes in early acorn developmental stages.

One putative *MYB* and one putative *MYB*-related transcripts, homologs of the Arabidopsis *MYB36* and *PRX72*, respectively, were specifically detected in the early and mid-stages of acorn development (cluster A and C, respectively). MYB transcription factors have diverse functions in plants including development, secondary metabolism, hormone signal transduction, disease resistance and abiotic stress tolerance [107, 108]. *MYB36* belongs to the *R2R3-MYB* gene family [109], which members have been described as the primary regulators of fruit flavonoid biosynthesis [110], among other functions [111]. Within the major flavonoid compounds present in flowers and fruits, the proanthocyanidins are astringent compounds that can offer protection during the early stages of fruit development against herbivory and pathogen attack [112, 113]. The specific presence of this transcript in the first stage of fruit development here analysed would be consistent with such a function in cork oak acorns, however, other roles should not be excluded. Our study also showed that the cork oak putative homolog to *AtPRX72* was expressed in a specific stage of acorn developmental, S3S4 (cluster C). This may be related to the significant increase in the size of the acorn from this point onwards, and therefore increased cell wall expansion processes, but it can be also due to an increase in hardiness of the fruit, suggesting that this gene may function in the lignification process in cork oak acorn, consistent with previously described roles [114–117].

Transcripts identified as specifically expressed in the last stages of development, included a putative transcription factor gene of the *FAR1* family, similar to the *FRS4*, which is a homolog of *FHY3*. Several studies report that FHY3 and FAR1 are required for regulating various aspects of plant processes, such as far-red-mediated seedling de-etiolation, the circadian clock, chloroplast division, and chlorophyll biosynthesis [118–124]. Tang *et al*. [125] have recently reported that knock-out mutants of FHY3 and/or FAR1 have reduced sensitivity to ABA-mediated inhibition of seed germination and seedling growth, lose water faster, and are less tolerant to drought stress than are wild type plants. Given the significance of drought tolerance traits for the successful cork oak natural regeneration process, it is tempting to consider that the putative homolog of *FRS4* in the cork oak mature acorn may be involved in conferring tolerance to water stress conditions. However, other roles are also possible [126].

Another family of proteins described as transcriptional regulators (http://plntfdb.bio.uni-potsdam.de/), the *OLEOSINS* (*OLEO1* and *OLEO4*) were also found specifically up-regulated in the last stages of the acorn development (S5 and S8). This is in accordance with the reported function of these plant specific genes in the control of oil body structure and accumulation of seed reserves, affecting seed germination and embryo phenotypes [127, 128]. Recent work in different species showed an increase in their accumulation during seed development [129–131].

Several members of two major families of TFs were present in all stages of the acorn development, bHLH and NAC. As an example of a putative bHLH TF gene, a homolog of the *Arabidopsis bHLH GLABRA3* (*GL3*), was up-regulated during the late stages of the acorn development (S5 and S8- cluster E) when the acorn pericarp starts to become visible out of the cupule and turning brown. If the function of this transcript is conserved, then it is tempting to speculate that its up-regulation during late acorn development is related to the regulation of anthocyanin biosynthesis [132, 133] that occurs during this phase, and that it may play an important function in seed dispersal by attracting herbivores. Putative NAC family members were also found along different stages of the acorn development. This family of TFs plays important roles in responses to plant biotic and abiotic stress [134, 135] but also in developmental processes such as seed and embryo development [135, 136]. Our data revealed three different up-regulated transcripts coding for the NAC protein family at the S1 developmental stage, two at S2 and S5 and one in both S5 and S8. One of these transcripts up-regulated in S1, *RD26*, has been mentioned above in relation to response to water [61, 63].

## Conclusions

In summary, our analysis allowed to cluster transcripts differentially expressed along acorn development in different profiles showing up-regulation in specific stages of development. While the DE transcripts putatively coding for transcriptional regulators associated to several biological processes were found up-regulated in given developmental stages throughout the whole acorn developmental process, other transcripts involved in specific processes such as response to water or carbohydrate metabolism were over-represented in particular stages. Future functional analysis of genes of interest identified in this work will be important to devise successful strategies for regeneration and breeding of this important species. This is especially important when considering the climate changes predicted for the Mediterranean region in the near future [137, 138] and the fact that drought is the main selection agent in Mediterranean ecosystems [21, 28]. Additionally, this dataset significantly contributes to increase the still scarce information on cork oak genomics providing tools for further molecular dissection of cork oak biology.

## Methods

### Plant material

Acorns were collected between mid June and late November 2009 from cork oak trees growing in private properties at six different locations in the South and Centre of Portugal: Quinta da Serra (Vila Nogueira de Azeitão), Alter do Chão, São Brás de Alportel, Monchique, Calhariz (Santarém) and Abrantes, after obtaining the required permissions. Samples were collected in accordance with the Portuguese legislation (Decree-laws n.° 169/2001 and n.° 155/2004) for cork oak. The term acorn is here used to refer the whole structure consisting of the pericarp and all the tissues enclosed by the pericarp including the seed. Acorns in the early stages of development (S1 to S4, Fig. 1a), were collected from 1 to 8 trees in each location and were immediately frozen in liquid nitrogen. To obtain the samples at S5 developmental stage and subsequent stages, branches bearing the acorns were kept at 4 ° C for up to 24 h before collecting the acorns and freezing in liquid nitrogen. In S8 stage samples, approximately 1/3 of the acorn part opposite to the embryo radicle was removed to minimize the presence of polysaccharides that could compromise the purity of isolated RNA. Additionally, some acorns from stage S3, S4, S5 and S8 were opened for embryo isolation (Fig. 1b); embryos from S8 stage were isolated by excising the embryo axis but excluding most cotyledonal tissue. In each collection, acorns and isolated embryos were carefully observed for evaluation and documentation of the developmental stage.

### RNA extraction

Frozen samples were first ground in a mill (M 20 Universal mill, Ika) previously cooled with liquid nitrogen and then transferred to a cooled mortar and reduced to a fine powder. Total RNA was isolated following a protocol described by Reid *et al*. with some minor modifications [139]. RNA isolation was performed separately by developmental stage, date of collection and mother tree.

Total RNA was purified using the RNeasy MinElute Cleanup kit (Qiagen) with on-column DNase I treatment (Qiagen RNase-Free DNase Set) and only samples with A_260/280_ > 1.8 were used for further steps. RNA integrity was assessed in 1 % (w/v) agarose gels after ethidium bromide staining and for a rigorous assessment of RNA quality, the RNA samples were run on a RNA Pico6000 chip in Agilent 2100 Bioanalyzer RNA (Agilent). Additionally, each sample was quantified by fluorescence with the Quant-iT Ribogreen RNA Assay kit (Invitrogen).

### Preparation of cDNA libraries and RNA-Seq

Two normalized and 5 non-normalized cDNA libraries were prepared. Normalized libraries were prepared with RNA isolated from acorn tissues or from isolated embryos, collected from a total of 5 and 3 trees, respectively. In each library, a pool of 2 μg total RNA containing equal amounts of RNA extracted from the several acorn (from S1 to S5, S7 and S8) or embryo developmental stages (from S3 to S5 and S8) was prepared. Double stranded cDNA was obtained using SMART technology [140] and the normalization was performed with the Duplex-Specific Nuclease (DSN) technology [141].

The 5 non-normalized cDNA libraries corresponded to different acorn developmental stages (S1, S2, S3 + S4, S5 and S8) collected from 7 different trees. For each library, a pool of total RNA was prepared containing 50 μg of total RNA. MicroPoly(A)Purist kit (Ambion) was used to isolate mRNA from each total RNA pool and 200 ng of mRNA were fragmented and used as template for double stranded cDNA production using cDNA Synthesis System Kit (Roche) followed by adaptor ligation.

Pyrosequencing of the normalized and non-normalized libraries was performed in the Titanium GS-FLX (454-Roche) at Biocant (Cantanhede, Portugal).

The data were deposited in the European Nucleotide Archive (ENA) under the accession number PRJEB6178/ERP005652. For each non-normalized library the accession numbers are the following: [ENA: ERX455655 for S1, ENA: ERX455656 for S2, ENA: ERX455657 for S3S4, ENA: ERX455658 for S5 and ENA: ERX455659 for S8]. For the normalized libraries [ENA: ERX455660 and ENA: ERX455661] are the accession numbers of the acorn and isolated embryo libraries, respectively.

### Reads pre-processing, de novo assembly and transcriptome annotation

The full workflow is schematized in the Additional file 16. First, the raw reads were filtered by SeqTrimNext [142] to remove adapter sequences and low quality/complexity sequences, which included fragments (window of 15 nts) with a quality value lower than 20, more than an 80 % of indeterminations, or 75 % of polyA or polyT sequences. Fragments with an E-value < 1e^-10^ and 85 % identity to contaminants such as plastids, mitochondria, ribosome and virus/bacteria sequences, were trimmed. Final sequences shorter than 40 nts were also excluded. RNA-Seq data was *de novo* assembled using MIRA version 3.4.0 [50] and Newbler version 2.6 [49]. MIRA was executed with the default 454 settings and without clipping steps. Newbler was executed with the default parameters. The individual assemblies were merged with CAP3 with default options and an identity threshold of 95 %.

Transcripts were compared with the Uniprot and Trembl databases using NCBI Blastx with an E-value of 1e^-6^. Only full-length plant proteins were included in the target database. “*Full Lengther Next*” scripts (https://rubygems.org/gems/full_lengther_next) were used to compare the aligned regions in query and target, in order to determine the right translation frame and classify the transcripts as complete, internal or terminal. These translated proteins constitute the *Q. suber* proteome used in subsequent comparisons. For those transcripts without any alignment, the program runs an Open Reading Frame (ORF) prediction step. Novel ORFs with a result higher than 0.7 (default threshold) were annotated as novel transcripts. The conserved motifs and structures in the sequences of these novel transcripts were identified by comparison against the motifs databases in EBI Interpro (http://www.ebi.ac.uk/interpro/interproscan.html). Transcripts were compared with the NCBI non-redundant (nr) and *Arabidopsis* TAIR protein databases using NCBI Blastx with an E-value of 1e^-10^. Results were imported in Blast2GO [143] to annotate the gene ontology terms, enzymatic protein codes and KEGG pathways. The reads of each transcript belonging to the same pathway were summed up. For each pathway, the number of reads in each stage was transformed in Z-scores, clustered, and plotted in a heatmap using Mayday [144]. Gene ontology terms and NCBI COGs (Clusters of Orthologous Groups of proteins) associated to each *Arabidopsis* gene were downloaded from TAIR (www.arabidopsis.org) and NCBI (ftp://ftp.ncbi.nih.gov/pub/COG/KOG/), and associated back to the original *Q. suber* transcript.

The transcriptomes of other Fagaceae species were downloaded from NCBI and Fagaceae Genomics Project (www.fagaceae.org). The proteome for each of them was built in a similar way as for *Q. suber* by comparison with the Uniprot and Trembl full plant proteins using the Full Lengther Next scripts. Proteomes were compared by pairs using NCBI Blastp. Proteins in a query species were considered as having an orthologous in a target species if they shared both a minimal identity and coverage of 70 %.

An alignment of the reads obtained from 19 normalized cork oak cDNA libraries prepared from different tissues and made available by [45], against the reads obtained from the normalized libraries described in this work, was performed to identify the transcripts specifically expressed in fruit and seed tissues. The *de novo* transcripts without aligned reads were considered as fruit and seed specific.

### Expression analysis and differentially expressed gene (DEG) clustering

The CLC Genomics Workbench (http://www.clcbio.com), version 6.7.2, was used to quantify the expression of the RNA-Seq data in four steps. Firstly, the reads from each of the five non-normalized libraries were aligned to the 80,357 contigs in the *Q. suber* assembly using the default scoring values and ignoring reads not uniquely mapping. Secondly, the number of aligned reads in each library was normalized by quartile normalization to take into account the different total number of reads per library. Third, a statistical analysis that compares the expected versus observed proportions of mapped reads by Kal’s z-test [145] implemented in CLC software between consecutive acorn developmental stages was used to identify the differentially expressed genes (DEGs). Finally, p-values were False Discovery Rate (FDR) corrected. Transcripts with a FDR value lower than 0.01 were considered as differentially expressed (DE).

DEGs were divided in 6 clusters according to the normalized number of aligned reads in each stage by Neural Gas clustering implemented in Mayday [144] based on Euclidian correlation between expression values. Neural Gas is an alternative to K-means clustering that resulted in clusters with a better balance of members. The list of transcripts in each cluster was used in Blast2GO to identify the enriched GO terms. Blast2GO enrichment analysis was based on a F-fisher test (FDR < 0.05). The relation among GO terms was assigned using REVIGO with the Resvik algorithm option [146] and R treemap library. DEGs annotated as related to response to water, including water transport and water deprivation were identified. We used the Plant Transcription Factor database (PLNTFDB, http://plntfdb.bio.uni-potsdam.de/) as reference to identify the TFs and other transcriptional regulators in our transcriptome. The database contains close to 30 K protein sequences of experimentally-identified elements from diverse plant species, and their classification in families according to their protein domains by HMM methods. The transcript sequences of the DEGs were aligned to the PLNTFDB using Blastx and a minimum E-value of 1e^-10^. We considered any transcript with a result under that threshold as a TF/transcriptional regulator, and annotated it within the family of the homologous with a lower E-value.

### Quantitative RT-PCR analysis

Reverse transcription quantitative real-time PCR (RT-qPCR) of a set of 20 DEGs was carried out to validate the expression profile obtained by 454 sequencing (Table 6). RNA samples were first treated with TURBO DNase (Ambion) and afterwards all cDNAs were synthesized from 1.5 μg of total RNA using the Transcriptor High Fidelity cDNA Synthesis Kit (Roche) with the anchored-oligo(dT)_18_ primers. Specific primers were designed using Geneious software [147]. Quantitative real-time PCR experiments were then performed in LightCycler 480 (Roche) using SYBR Green I Master (Roche) and 96-well plates. For the genes tested, 3 biological replicates were used and the reaction mixtures were performed in a final volume of 16 μl containing 8 μl of 2× SYBR Green I Master, 400 nM of each primer and 1.5 μl of cDNA as template. The amplification program was the same for all genes tested: 95 ° C for 10 min, 45 cycles of 10 s at 95 ° C, 20 s at 60 ° C and 10 s at 72 ° C, except for *β-AMYLASE 1* (*BAM1*), *GALACTINOL SYNTHASE 2* (*GolS2*) and *CALCIUM DEPENDENT PROTEIN KINASE 10* (*CPK10*) for which the annealing temperature was 62 ° C. A calibrator sample was used in each plate to normalize the values obtained and the potential differences among plates. Normalization was carried out with two reference genes *ACTIN* (*ACT*) and *CLATHRIN ADAPTOR COMPLEXES* (*CAC*) [139]. Normalized relative quantities were obtained through the formula $NRQ=\frac{{E_{goi}}^{\varLambda Ct,goi}}{f\sqrt{{\displaystyle {\prod}_{\mathrm{o}}^f{E}_{re{f}_{\mathrm{o}}}^{\varDelta Ct,re{f}_{\mathrm{o}}}}}}$, where E is the efficiency of the amplification for each primer pair in each tissue, *f* the number of reference genes used to normalize the data, *goi* and *ref* are the gene of interest and the reference gene, respectively, and ΔCt is the Ct of the calibrator minus the Ct value of the sample in test [148–150]. The data obtained from the RNA-Seq experiment and the RT-qPCR were compared. From the RNA-Seq a logarithmic ratio of base 2 between the counts of a gene in each developmental stage and the mean counts of the same gene in all developmental stages were made. A similar approach was followed for the data obtained by RT-qPCR by doing a logarithmic ratio of base 2 between the normalized quantities of the gene of interest in each developmental stage and the mean normalized quantities of the same gene in all developmental stages in analysis. For genes where the RNA-Seq values were zero in some of the developmental stages, a value of 1 was added to all the RNA-Seq results of those genes to avoid dividing by zero [151].

### Availability of supporting data

The data sets supporting the results of this article are available in the European Nucleotide Archive (ENA) under the accession number PRJEB6178, http://www.ebi.ac.uk/ena/data/view/PRJEB6178. The reads libraries are also available in the ENA repository, http://www.ebi.ac.uk/ena/data/view/ERX455655-ERX455661. The cork oak transcriptome assembly performed in this work is directly available in ENA, http://www.ebi.ac.uk/ena/data/view/HABZ01000000, as well as each of the individual contigs, http://www.ebi.ac.uk/ena/data/view/HABZ01000001-HABZ01080357.

## Additional files

Additional file 1: File S1.
*Quercus suber* transcript classification and predicted function. Annotation and classification of the assembled transcripts using Full Lengther Next scripts and functional annotation using the Uniprot database. Annotation and characterization (against the motifs databases from EBI Interpro) of the transcripts specific to the cork oak acorn. Additional file 2: Table S1. Comparison of the transcriptomes of several *Fagaceae* tree species with full length plant proteins in UniprotKB. Transcriptomes were retrieved from the UniprotKB database. Additional file 3: Table S2. Identification of shared proteins among some members of the *Fagaceae* family. Sequences from a query species (rows) with homologous in a different target species (columns). Proteins were considered homologous between the query and target species if they share a minimal identity and coverage of 70 %. Additional file 4: Figure S1. Quality assessment of the gene annotation. (A) Species with the best BLASTX alignment of each query sequence. (B) Values of similitude from Blastx alignments. (C) Number of gene ontology (GO) terms per sequence length. Additional file 5: File S2. Functional annotation of *Quercus suber* transcripts. Annotation was based on the homologous in NCBI and *Arabidopsis* (TAIR) databases using Blastx with an E-value of 1e^-10^ and results were submitted in Blast2GO to annotate gene ontology (GO) terms. Additional file 6 File S3. Classification of Orthologous Groups (COG). Analysis of the COGs present in data retrieved from *Q. suber*, CODB (Cork oak Database), *Q. petraea* and *Q. robur* and association to each *Arabidopsis* gene. Additional file 7: Figure S2. Distribution of Clusters of Orthologs Groups (COGs) between different species of the *Quercus* genus: *Q. suber*, *Q. petraea* and *Q. robur* and the data from the Cork Oak Database (CODB). Additional file 8: Table S3. Biological domain of the clusters of Orthologous Groups of proteins (COGs) specific to *Q. suber*. Additional file 9: File S4. Number of cork oak transcripts in KEGG level 2 and 3 pathways. Expression of each pathway was quantified as the sum of the counts (mapped reads) of all the transcripts belonging to it. Additional file 10: Figure S3. Venn diagram showing the number of transcripts expressed in the different acorn developmental stages. Additional file 11: File S5. Identification of the genes differentially expressed during acorn development by Z-test. Results of the clustering, fold-change and functional annotation (NCBI, TAIR, UNIPROT) of the differentially expressed genes (DEGs) in consecutive stages of acorn development. Annotation of the DEGs specific to the cork oak acorn. Additional file 12: File S6. Enrichment analysis of the differentially expressed genes (DEGs). Comparison of the DEGs with the complete transcriptome evidencing the gene ontology (GO) terms over-represented in all the clusters and separately in each cluster. Additional file 13: Figure S4. Treemap of the GO terms from the DEGs. The area of each cell is proportional to the number of genes annotated with that term. Additional file 14: File S7. Differentially expressed genes related to response to water, water deprivation or water transport. Cluster annotation, Uniprot locus, association with *Arabidopis* homologs, normalized counts in each acorn developmental stage, fold-change between consecutive stages, stage expression factor (SEF) and contigs specific to each cluster. Additional file 15: File S8. Differentially expressed genes annotated as transcription factors and transcriptional regulators. Cluster annotation, Uniprot locus, association with *Arabidopis* homologs, normalized counts in each acorn developmental stage, fold-change between consecutive stages, stage expression factor (SEF) and contigs specific to each cluster. Additional file 16: Figure S5. Workflow of the 454 sequencing data analyses. After sequencing, the raw reads obtained were pre-processed to remove non desired sequences, including low quality sequences and contaminants. The clean reads from all libraries were then assembled using two different assemblers and then joined using CAP3. The obtained contigs that were then associated to gene ontology (GO) terms and searched for transcription factors. After mapping the differentially expressed genes (DEGs) along cork oak acorn development were identified.

## Abbreviations

DE: Differentially expressed
DEGs: Differentially expressed genes
ESTs: Expressed sequence tags
TF: Transcription factor.
