# Supplementary material for: Characterization of the cork oak transcriptome dynamics during acorn development
Source: BMC Plant Biol. 2015 Jun 25;15:158. doi: 10.1186/s12870-015-0534-1 (PMC4479327; doi:10.1186/s12870-015-0534-1)
Supplement: Additional file 2: Table S1. — Comparison of the transcriptomes of several Fagaceae tree species with full length plant proteins in UniprotKB. Transcriptomes were retrieved from the UniprotKB database. [file 12870_2015_534_MOESM2_ESM.pdf]

	Q. suber	***Q. robur***	***Q. petraea***	***Q. rubra***	***Q. alba***	***F. sylvatica***	***C. mollisima***	***C. dentata***
Total contigs	80357	81671	58230	28041	22102	31309	48335	45288
Translated proteins	56517 (70.3%)	65030 (79.6%)	47567 (81.7%)	20186 (72.0%)	16200 (73.3%)	27765 (88.7%)	31907 (66.0%)	31338 (69.2%)
Unique Uniprot homologous	24474 (30.5%)	17565 (21.5%)	14618 (25.1%)	13804 (49.2%)	11777 (53.3%)	10264 (32.8%)	19723 (40.8%)	18814 (41.5%)
Complete proteins	19146 (33.9%)	16112 (24.8%)	9481 (19.9%)	1864 (9.2%)	1401 (8.6%)	3830 (13.8%)	4947 (15.5%)	2392 (7.6%)
C-terminus proteins	11410 (20.2%)	21881 (33.6%)	16121 (33.9%)	4477 (22.2%)	3843 (23.7%)	10544 (38.0%)	7252 (22.7%)	6668 (21.3%)
N-terminus proteins	10108 (17.9%)	14922 (22.9%)	12361 (26.0%)	3570 (17.7%)	2650 (16.4%)	7299 (26.3%)	5314 (16.7%)	5970 (19.1%)
Internal proteins	15509 (27.4%)	12091 (18.6%)	9584 (20.1%)	10218 (50.6%)	8258 (51.0%)	6086 (21.9%)	14200 (44.5%)	16252 (51.9%)
Misassembled	344 (0.6%)	24 (0.04%)	20 (0.04%)	57 (0.3%)	48 (0.3%)	6 (0.02%)	194 (0.6%)	56 (0.2%)
