# Supplementary material for: Characterization of the cork oak transcriptome dynamics during acorn development
Source: BMC Plant Biol. 2015 Jun 25;15:158. doi: 10.1186/s12870-015-0534-1 (PMC4479327; doi:10.1186/s12870-015-0534-1)
Supplement: Additional file 3: Table S2. — Identification of shared proteins among some members of the Fagaceae family. Sequences from a query species (rows) with homologous in a different target species (columns). Proteins were considered homologous between the query and target species if they share a minimal identity and coverage of 70 %. [file 12870_2015_534_MOESM3_ESM.pdf]

**% and total**	**TOTAL***	***Q. suber***	***Q. robur***	***Q. petraea***	***Q. rubra***	***Q. alba***	***F. sylvatica***	***C. mollisima***	***C. dentata***	**AVERAGE****
Q. suber	56163	100<br>56163	67.8<br>38071	61.8<br>34704	65.6<br>36821	59.0<br>33145	50.7<br>28480	80.9<br>45433	80.5<br>45239	66.6
Q.robur	65006	94.4<br>61349	99.9<br>64925	84.2<br>54713	83.1<br>54048	79.3<br>51536	68.2<br>44355	92.1<br>59865	91.7<br>59628	84.7
Q. petraea	47547	96.2<br>45718	88.1<br>41873	99.9<br>47492	82.6<br>39288	79.3<br>37681	69.1<br>32854	92.9<br>44177	91.5<br>43519	85.7
Q. rubra	20129	94.7<br>19061	67.2<br>13529	60.3<br>12145	99.9<br>20113	60.3<br>12131	48.0<br>9654	86.2<br>17360	84.1<br>16922	71.5
Q. alba	16152	95.2<br>15384	70.4<br>11379	63.9<br>10327	72.0<br>11628	99.9<br>16134	50.5<br>8150	87.1<br>14074	80.8<br>13053	74.3
F. sylvatica	27759	93.0<br>25815	77.7<br>21566	74.0<br>20545	75.9<br>21082	71.8<br>19920	100<br>27747	85.7<br>23786	82.0<br>22765	80.0
C. mollisima	31713	92.4<br>29289	61.6<br>19537	55.4<br>17555	59.0<br>18726	51.5<br>16219	42.8<br>13574	99.9<br>31686	73.2<br>23219	62.3
C. dentata	31282	93.7<br>29308	63.8<br>19971	56.5<br>17688	62.2<br>19452	53.5<br>16741	44.4<br>13901	84.9<br>26550	99.9<br>31244	65.6
AVERAGE**		94.2	70.9	65.2	71.5	65.0	53.4	87.1	83.4	

*Translated proteins minus misassembled proteins

**Average excluding species own value.
