# Supplementary figures and images for: Characterization of the cork oak transcriptome dynamics during acorn development
Source: BMC Plant Biol. 2015 Jun 25;15:158. doi: 10.1186/s12870-015-0534-1 (PMC4479327; doi:10.1186/s12870-015-0534-1)

A

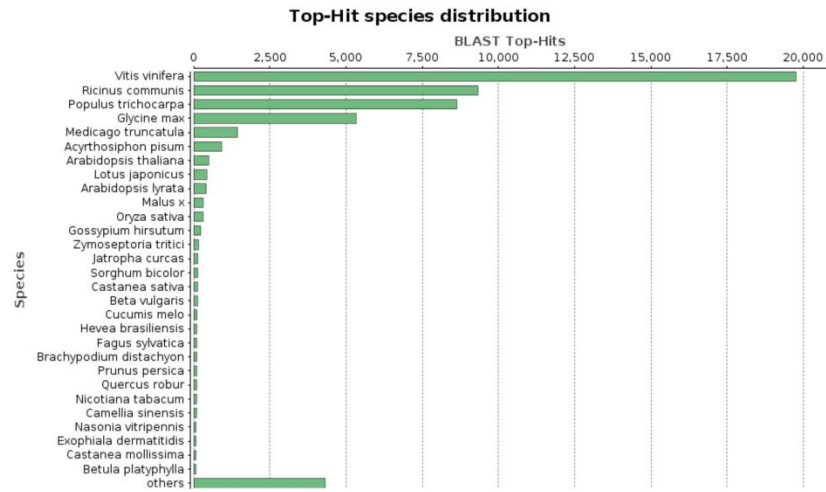

B

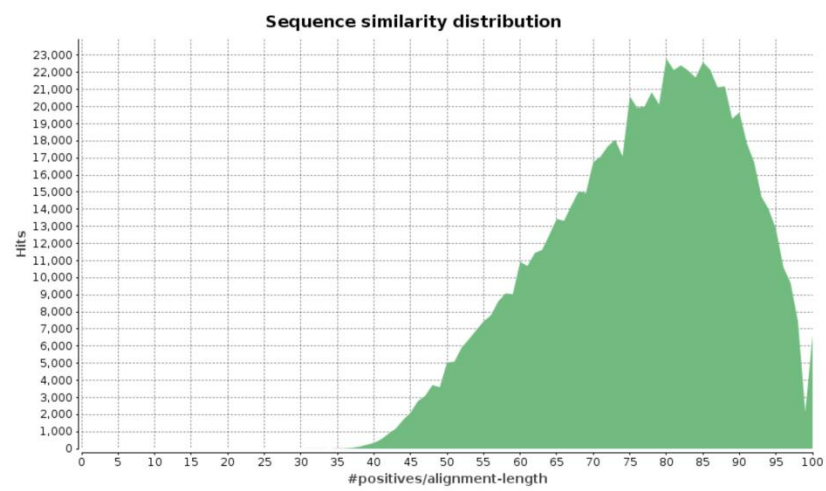

C

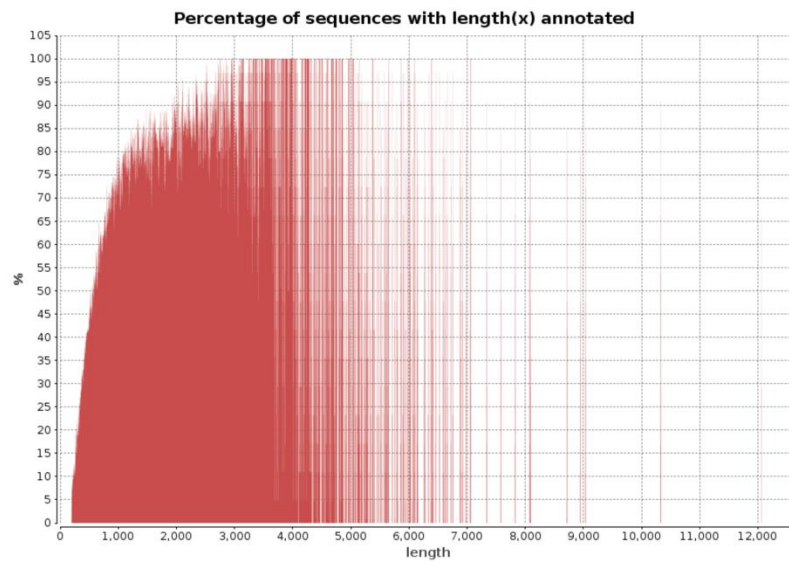

Supplement: Additional file 4: Figure S1. — Quality assessment of the gene annotation. (A) Species with the best BLASTX alignment of each query sequence. (B) Values of similitude from Blastx alignments. (C) Number of gene ontology (GO) terms per sequence length. [file 12870_2015_534_MOESM4_ESM.pdf]

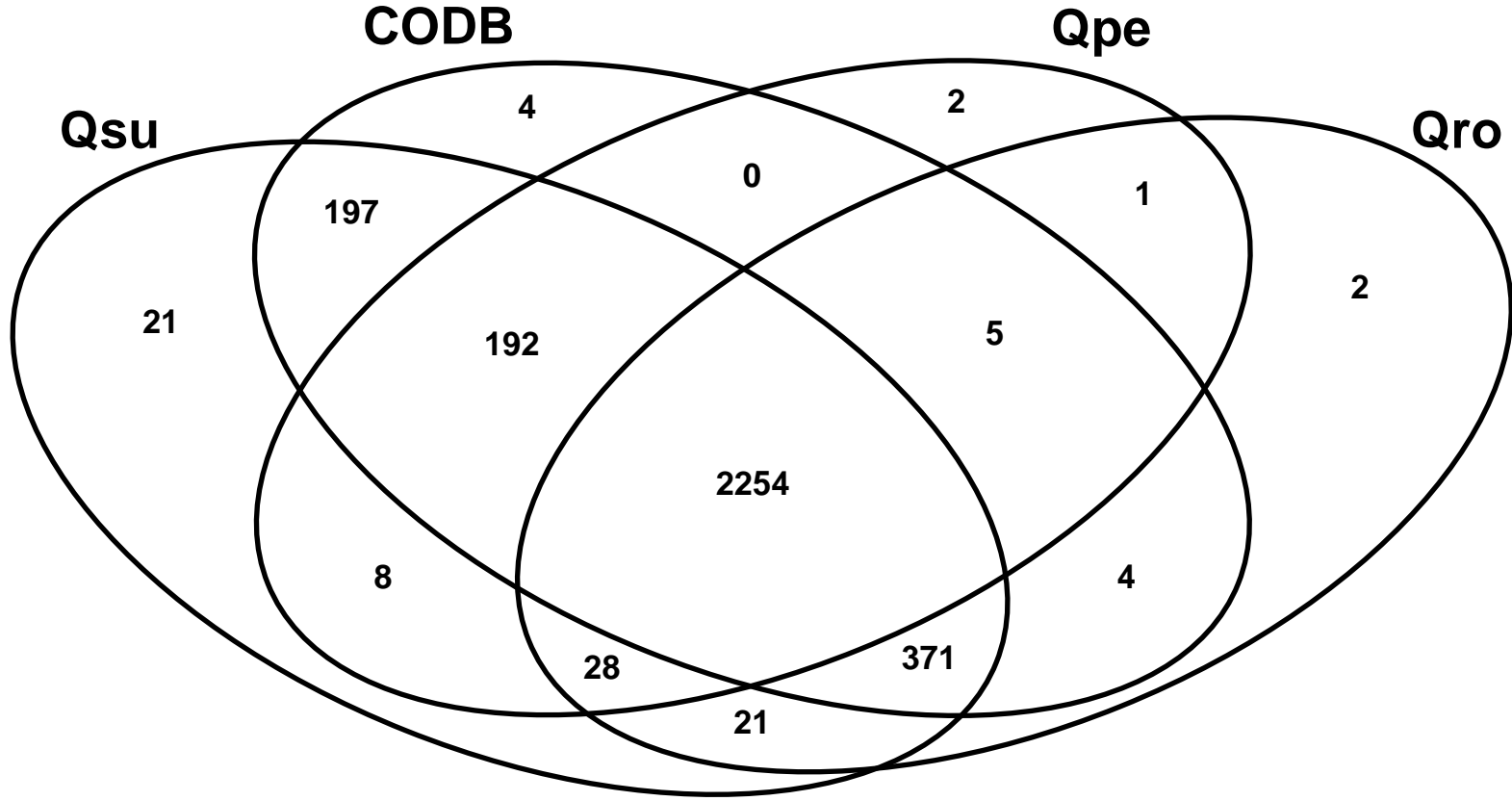

Supplement: Additional file 7: Figure S2. — Distribution of Clusters of Orthologs Groups (COGs) between different species of the Quercus genus: Q. suber, Q. petraea and Q. robur and the data from the Cork Oak Database (CODB). [file 12870_2015_534_MOESM7_ESM.pdf]

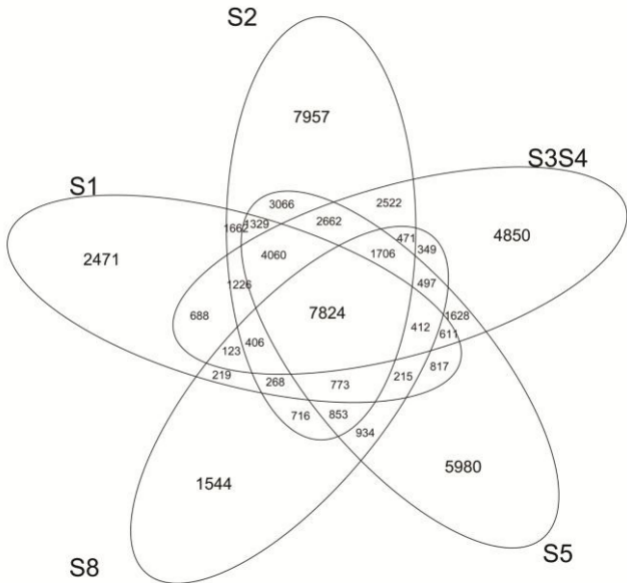

Supplement: Additional file 10: Figure S3. — Venn diagram showing the number of transcripts expressed in the different acorn developmental stages. [file 12870_2015_534_MOESM10_ESM.pdf]

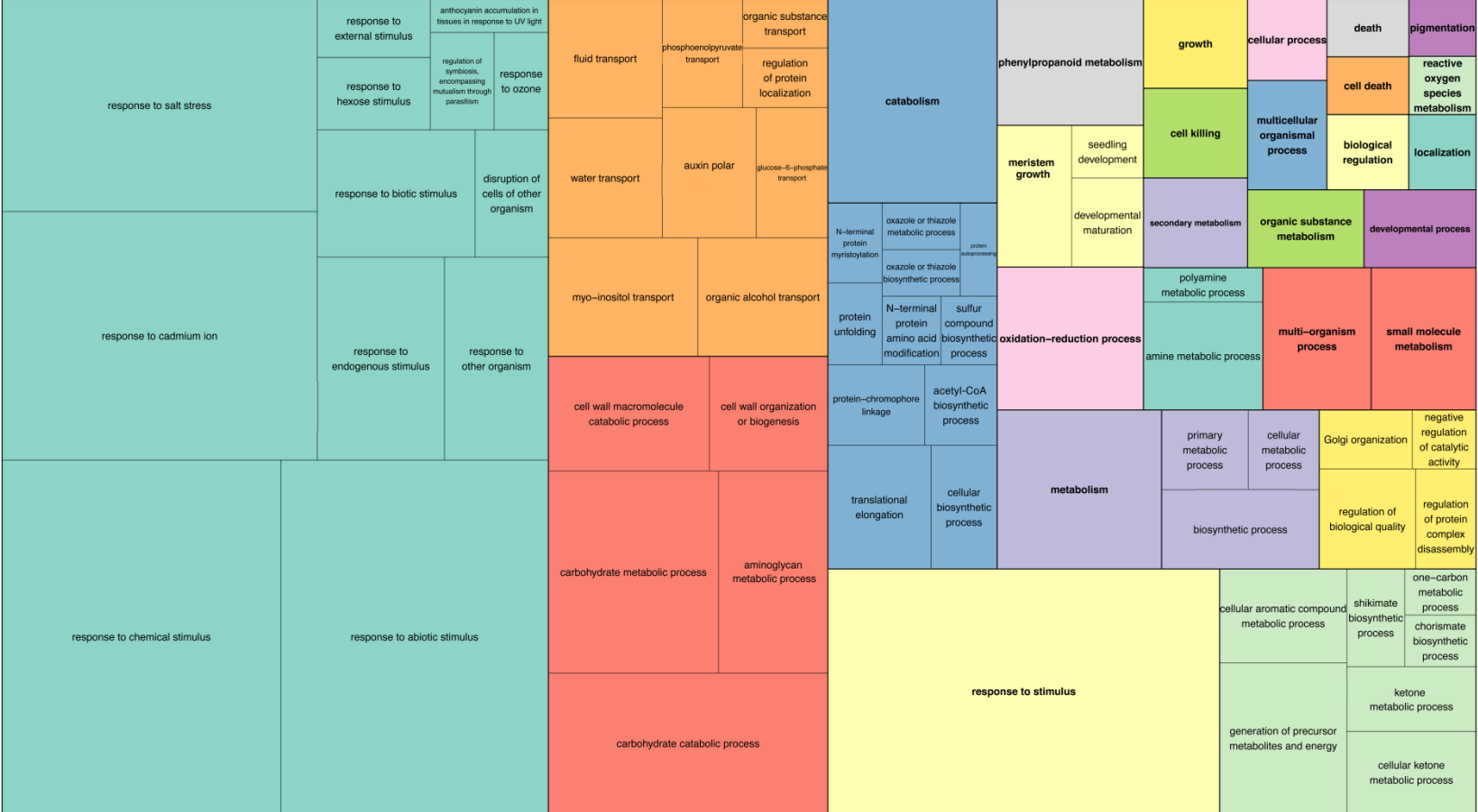

Supplement: Additional file 13: Figure S4. — Treemap of the GO terms from the DEGs. The area of each cell is proportional to the number of genes annotated with that term. [file 12870_2015_534_MOESM13_ESM.pdf]

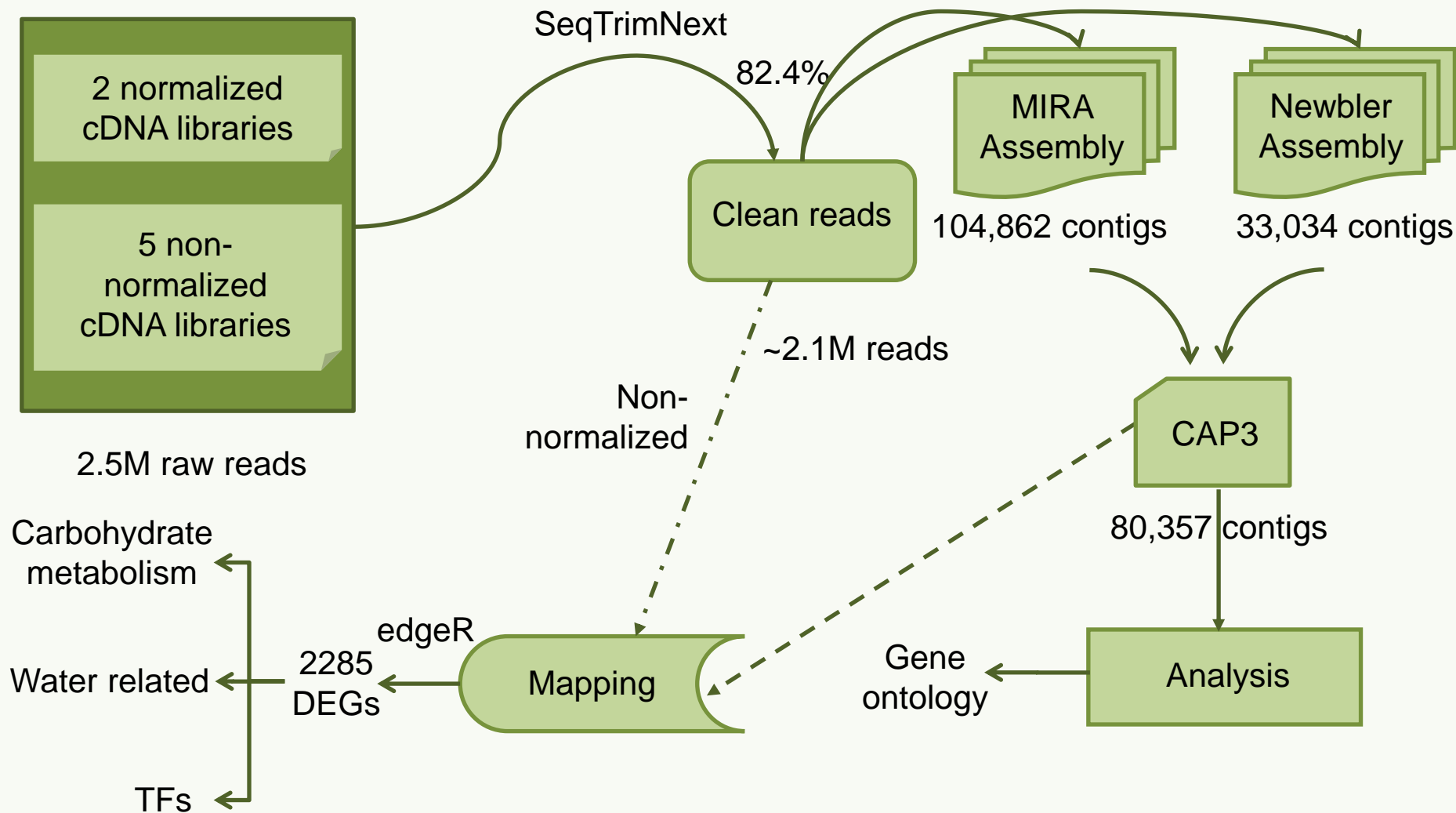

Supplement: Additional file 16: Figure S5. — Workflow of the 454 sequencing data analyses. After sequencing, the raw reads obtained were pre-processed to remove non desired sequences, including low quality sequences and contaminants. The clean reads from all libraries were then assembled using two different assemblers and then joined using CAP3. The obtained contigs that were then associated to gene ontology (GO) terms and searched for transcription factors. After mapping the differentially expressed genes (DEGs) along cork oak acorn development were identified. [file 12870_2015_534_MOESM16_ESM.pdf]
