# Supplementary material for: Characterization of the cork oak transcriptome dynamics during acorn development
Source: BMC Plant Biol. 2015 Jun 25;15:158. doi: 10.1186/s12870-015-0534-1 (PMC4479327; doi:10.1186/s12870-015-0534-1)
Supplement: Additional file 8: Table S3. — Biological domain of the clusters of Orthologous Groups of proteins (COGs) specific to Q. suber. [file 12870_2015_534_MOESM8_ESM.pdf]

Group	COGs	%
A - RNA processing and modification	14	6.31
B - Chromatin structure and dynamics	4	1.80
C - Energy production and conversion	4	1.80
D - Cell cycle control, cell division, chromosome partitioning	12	5.41
E - Amino acid transport and metabolism	4	1.80
F - Nucleotide transport and metabolism	3	1.35
G - Carbohydrate transport and metabolism	2	0.90
H - Coenzyme transport and metabolism	5	2.25
I - Lipid transport and metabolism	11	4.95
J - Translation, ribosomal structure and biogenesis	14	6.31
K - Transcription	11	4.95
L - Replication, recombination and repair	27	12.16
M - Cell wall/membrane/envelope biogenesis	2	0.90
O - Post-translational modification, protein turnover, and chaperones	11	4.95
P - Inorganic ion transport and metabolism	2	0.90
Q - Secondary metabolites biosynthesis, transport, and catabolism	1	0.45
R - General function prediction only	29	13.06
S - Function unknown	46	20.72
T - Signal transduction mechanisms	8	3.60
U - Intracellular trafficking, secretion, and vesicular transport	6	2.70
Y - Nuclear structure	3	1.35
Z - Cytoskeleton	3	1.35
	222	100.00
